# Running Exercise Promotes Astrocyte-Mediated Structural Plasticity in the Amygdalar BLA and CeA to Alleviate Anhedonia-like Behavior Alterations

**DOI:** 10.3390/cells15080693

**Published:** 2026-04-14

**Authors:** Xinyan Ren, Yanmin Luo, Qian Xiao, Jing Li, Yuning Zhou, Yuhui Deng, Xingyu Wu, Huifang Luo, Yue Li, Lin Jiang, Chunni Zhou, Dujuan Huang, Xiaoyun Dou, Fenglei Chao, Lei Zhang, Xin Liang, Yong Tang, Jing Tang

**Affiliations:** 1 Department of Histology and Embryology, School of Basic Medical Sciences, Chongqing Medical University, Chongqing 400016, China; 2 Laboratory of Stem Cell and Tissue Engineering, School of Basic Medical Sciences, Chongqing Medical University, Chongqing 400016, China; 3 Department of Physiology, School of Basic Medical Sciences, Chongqing Medical University, Chongqing 400016, China; 4 Department of Radioactive Medicine, School of Basic Medical Sciences, Chongqing Medical University, Chongqing 400016, China; 5 Lab Teaching and Management Center, School of Basic Medical Sciences, Chongqing Medical University, Chongqing 400016, China; 6 Institute of Life Science, Chongqing Medical University, Chongqing 400016, China; 7 Chongqing Medical University Science and Technology Innovation Center, Chongqing Medical University, Chongqing 400016, China; 8 Department of Pathology, School of Basic Medical Sciences, Chongqing Medical University, Chongqing 400016, China

**Keywords:** astrocyte, amygdala, chronic unpredictable stress, anhedonia-like behavior, running exercise, stereology, astrocyte proliferation, synaptic remodeling, PSD95

## Abstract

**Highlights:**

**What are the main findings?**

- Unbiased stereological analysis shows that while chronic unpredictable stress (CUS) has no significant effect on the BLA or CeA volume, running exercise selectively increases CeA volume in rats.
- Running exercise reverses CUS-induced reductions in astrocyte number, proliferation, and morphological complexity in both BLA and CeA. Running exercise increases the number of excitatory synaptic contacts (PSD95^+^ puncta) associated with astrocytes in the amygdala of chronically stressed rats.

**What are the implications of the main findings?**

- These findings highlight that astrocyte-mediated structural remodeling in amygdala subregions is an effective target for the antidepressant effects of exercise.
- Astrocyte plasticity and excitatory synapse maintenance in amygdalar subregions may represent a potential target for therapeutic intervention in stress-related affective disorders.

**Abstract:**

Amygdala dysfunction is implicated in stress-related affective disorders, and astrocytes are key regulators of amygdalar neuroplasticity. Here, we examined whether running exercise modulates astrocyte number, morphology, proliferation, and excitatory synaptic contacts in the basolateral amygdala (BLA) and central amygdala (CeA) in rats exposed to chronic unpredictable stress (CUS). Anhedonia-like behaviors were evaluated using the sucrose preference test, while anxiety-related behaviors were assessed using the elevated plus maze and open field tests. Unbiased stereological three-dimensional quantification was used to assess amygdalar volume and estimate astrocyte numbers in BLA and CeA, and immunofluorescence with morphological reconstruction was performed to quantify astrocytic complexity, proliferation, and astrocyte-associated PSD95^+^ puncta. Running exercise significantly increased sucrose preference in CUS rats, whereas elevated plus maze and open field measures were not significantly changed. CUS reduced astrocyte number and proliferation, and induced astrocytic morphological atrophy in both subregions. These alterations were reversed by running. Moreover, running increased the number of excitatory synapses contacted by astrocytes in the BLA and CeA of CUS rats. These findings suggest that running promotes astrocyte-mediated structural remodeling in amygdalar subregions, which may contribute to the regulation of anhedonia-like behavioral alterations associated with chronic stress.

## 1. Introduction

Depression is a common and disabling mental disorder affecting more than 300 million people worldwide [1,2], resulting in a substantial socioeconomic burden [3]. Its main clinical features include persistent low mood, anhedonia, and cognitive dysfunction [4,5]. Although the monoamine hypothesis has long dominated the understanding of depression, it does not fully account for the dynamic and complex alterations in signaling and neural adaptability observed in the disorder [6,7,8]. Accordingly, increasing attention has shifted toward impaired neuroplasticity underlying specific behavioral dimensions of depression such as anhedonia and stress-related affective alterations [9,10,11].

As a key node of the limbic system, the amygdala is widely recognized as the “center of fear and emotion” and plays an essential role in emotional processing [12,13,14,15,16]. Its functions are mediated by heterogeneous subnuclei. The basolateral amygdala (BLA) serves as a higher-order input hub that receives and integrates sensory information from cortical and subcortical regions and supports emotional learning and memory [15,17,18]. In contrast, the central amygdala (CeA) acts as the principal output nucleus, relaying signals from the BLA and other regions to the hypothalamus and brainstem to regulate autonomic and behavioral emotional responses [19,20]. Both functional and structural abnormalities of the amygdala have been identified as core neuropathological features of depression and related affective symptoms. Functionally, patients with depression often exhibit amygdala hyperactivity, particularly heightened responses to negative emotional stimuli such as fearful faces, along with molecular abnormalities, including disrupted amino acid signaling and altered gene expression [21,22,23]. Structurally, studies have reported alterations in amygdala volume. Although the results show some heterogeneity, possibly related to factors such as age of onset [24] or comorbidity with anxiety [25], and vary in clinical relevance, most evidence indicates abnormal amygdala volume, with reductions observed in first-episode depression patients that correlate with symptom severity [26]. Moreover, effective antidepressant treatments such as electroconvulsive therapy, can increase amygdala subregion volume [27], suggesting its potential reversibility. Together, these findings indicate that amygdala dysfunction contributes critically to emotional dysregulation associated with depression-related behaviors.

Historically, research on depression has primarily focused on neurons [28,29,30]. However, astrocytes are increasingly recognized as active regulators of neural circuit function by maintaining synaptic homeostasis [31], forming “tripartite synapses” [32], and secreting neurotrophic factors. Postmortem [33] and animal [34] studies have shown that astrocytes in emotion-related brain regions, including the prefrontal cortex and hippocampus, often exhibit reduced density, morphological atrophy, and functional impairment [35,36,37]. Emerging evidence also supports decreased astrocyte density and structural changes in the amygdala in major depression and under chronic stress [33,38]. Notably, targeted regeneration of new astrocytes in the adult mouse prefrontal cortex can reverse stress-induced behavioral alterations within two weeks, and this regenerative capacity is preserved even under depressive conditions [39]. Additional evidence suggests that restoring astrocyte function through pathways involving neuroinflammatory signaling (IL-6/IL-R) [40] or non-coding RNAs (e.g., circHIPK2) [41] effectively ameliorates anhedonia-like behaviors. Despite these advances, astrocytic alterations within distinct amygdala subregions remain incompletely defined, particularly regarding astrocyte proliferation/renewal dynamics and their influence on synaptic microenvironments during chronic stress.

Running exercise is an effective non-pharmacological intervention for depression [42,43], and its therapeutic effects are closely associated with enhanced neuroplasticity [40,44], with astrocytes recognized as key cellular targets [44,45]. Exercise has been shown to promote astrocyte proliferation in the hippocampus and to reverse stress-induced astrocyte loss [45], suggesting a potential cellular mechanism underlying exercise-induced resilience to stress. However, whether and how astrocytes in functionally distinct amygdala subregions (BLA and CeA) are altered by chronic stress and restored by running exercise remain unclear. In particular, the dynamics of newly proliferating astrocytes and the extent to which astrocytes remodel local excitatory synaptic contacts in these subregions have not been well-characterized.

Therefore, in this study, chronic unpredictable stress (CUS) was used to induce stress-related behavioral alterations, with a particular focus on anhedonia-like behavior, in rats. The effects of CUS and running exercise were evaluated using the sucrose preference test (SPT), elevated plus maze (EPM), and open field test (OFT). Unbiased stereological techniques, immunofluorescence, and three-dimensional reconstruction were utilized to assess how running exercise influences astrocyte number, morphology, proliferation, and astrocyte-associated excitatory synapses within the BLA and CeA. We show that running exercise reverses CUS-induced anhedonia-like behavioral deficits and amygdalar atrophy, restores astrocyte abundance and morphological complexity, and is accompanied by the recovery of astrocyte-associated excitatory synaptic contacts in both subregions, highlighting an astrocyte-mediated structural plasticity mechanism that may contribute to the regulation of anhedonia-like behaviors.

## 2. Materials and Methods

### 2.1. Animals

Forty-five male Sprague–Dawley rats (4–6 weeks old, 150 ± 10 g) were obtained from Chongqing Medical University. Animals were housed in polycarbonate cages with autoclaved bedding under controlled conditions (22 ± 2 °C, 50 ± 5% humidity, 12 h/12 h light–dark cycle, lights on at 07:00). Food and water were provided ad libitum. After 7 days of acclimation (4–5 per cage), rats were randomly assigned to either a control group (CON, *n* = 15) or a chronic unpredictable stress group (CUS, *n* = 30). In this research, all rat experiments were performed according to the National Institutes of Health Guide for the Care and Use of Laboratory Animals (NIH Publication No. 85-23). All experiments were performed blindly and approved by the Ethics Committee of Chongqing Medical University (approval No. 2021067).

### 2.2. CUS Intervention and Experimental Timeline

The CUS group was exposed to two different stressors per day for five weeks. Stressors were randomly selected from thermal challenge, light/dark disruption, noise, food or water deprivation, empty bottle, damp bedding, foot shock, restraint, tail pinch, and cage tilt. Control rats were maintained under standard housing conditions without stress exposure. Sucrose preference and body weight were measured weekly during the CUS period. After the completion of CUS, CUS rats were randomly divided into a sedentary subgroup (the CUS group, *n* = 15) and a running exercise subgroup (the CUS+running group, *n* = 15). The control group remained sedentary throughout the experiment [46,47].

### 2.3. Behavioral Testing

Anhedonia-like behaviors were assessed using the sucrose preference test (SPT), elevated plus maze (EPM), and open field test (OFT). All behavioral tests were performed by investigators blinded to group allocation [48].

### 2.4. Perfusion and Tissue Preparation

All subsequent procedures and analyses were performed under blinded conditions. Five rats were randomly selected from each group for stereological analysis. Animals were deeply anesthetized with 1% sodium pentobarbital (4 mL/kg, i.p.) and transcardially perfused with 4% paraformaldehyde (PFA) in 0.1 M phosphate-buffered saline (PBS, pH 7.4).

Following perfusion fixation, the brains were removed, and the meninges, cerebellum, and brainstem were carefully removed. One cerebral hemisphere (left or right) was randomly selected for subsequent analyses. The selected hemispheres were post-fixed in 4% PFA at 4 °C for at least 24 h and subsequently cryoprotected in graded sucrose solutions (10–30% in PBS) at 4 °C until the tissue sank.

**Figure 1:**
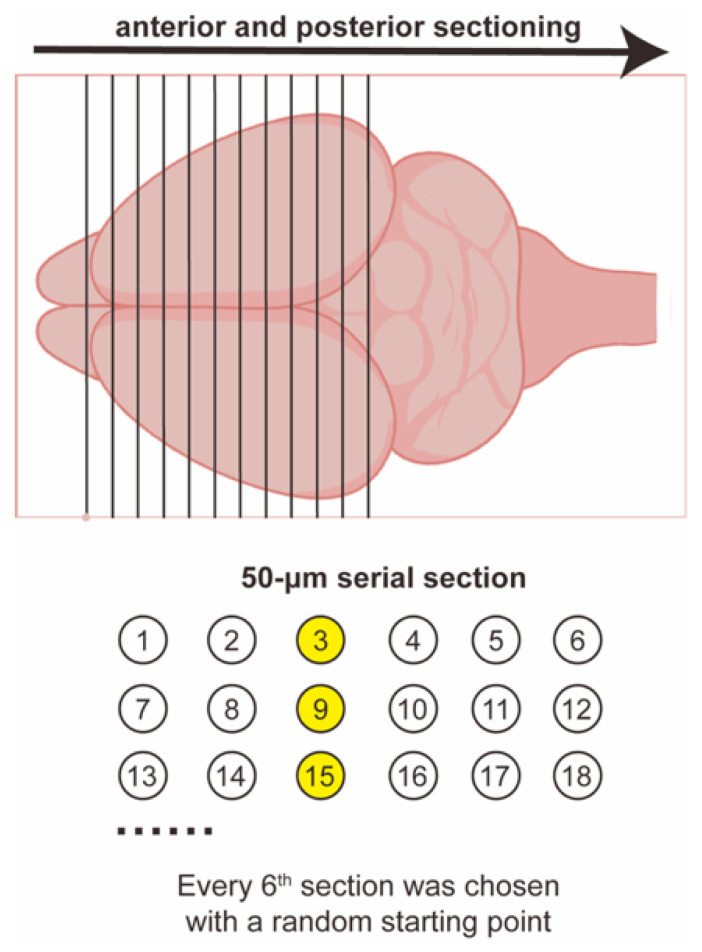
Systematic random sampling of serial coronal brain sections for stereological analysis. Serial coronal sections (50 μm thick) were collected along the anterior–posterior axis and systematically divided into six parallel series. With a random starting point, every sixth section was selected for analysis (section sampling fraction, *ssf* = 1/6). For example, sections 3, 9, and 15 (highlighted in yellow) were chosen. This approach ensured systematic and unbiased sampling across the entire amygdala. Two series were randomly selected from the six section series for subsequent Nissl staining and GFAP immunohistochemistry.

The hemispheres were then frozen at −60 °C and coronally sectioned at a thickness of 50 μm using a cryostat (Leica CM1860, Wetzlar, Germany). Serial sections were collected using a systematic random sampling strategy, with every sixth section selected for analysis (section sampling fraction, *ssf* = 1/6). Serial sections were collected using a systematic random sampling strategy (Figure 1). On average, approximately 12 sections spanning the amygdala were obtained from each hemisphere.

Prior to storage, sections were rinsed in PBS and ethanol and stored at −20 °C until further processing. For subsequent Nissl staining and GFAP immunohistochemistry, two series were randomly selected from the six serially collected section sets for each brain.

### 2.5. Toluidine Blue (Nissl) Staining and Amygdala Volume Estimation

**Figure 2:**
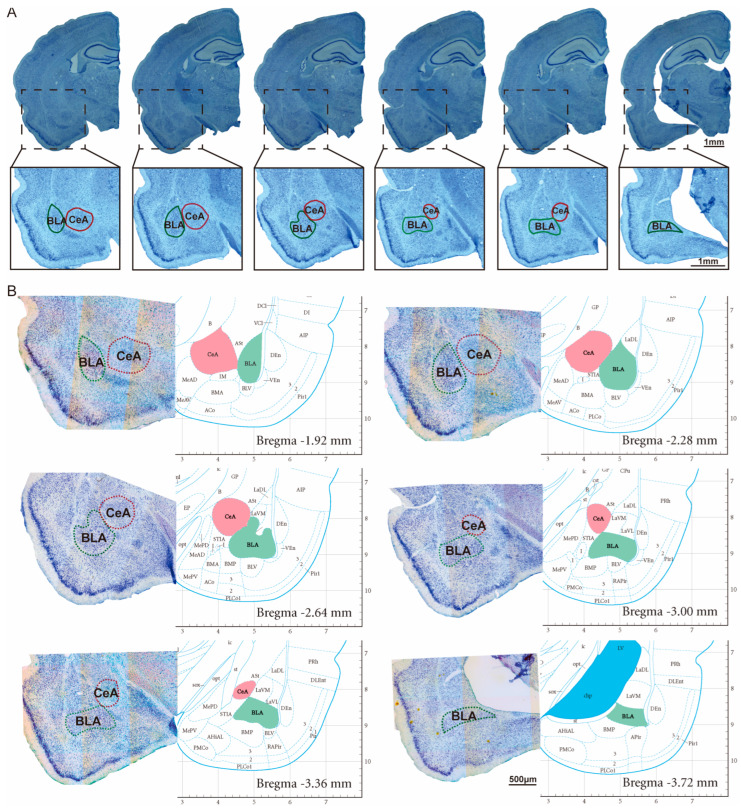
Cytoarchitectonic delineation and stereological volume estimation of the amygdala using Nissl staining. (**A**) Representative serial coronal sections illustrating the anterior–posterior extent of the amygdala. Nissl-stained images are shown at low (1.25×) and higher (2.5×) magnification, with BLA (green) and CeA (red) outlined. Scale bar: 1 mm. (**B**) Cytoarchitectonic criteria used for the delineation of amygdala subregions. The six panels correspond one-to-one to the six sections shown in (**A**). For each level (Bregma −1.92 mm to −3.72 mm), a representative higher-magnification Nissl-stained image (10×; left) reveals neuronal cell bands and regional differences in neuronal density. These cytoarchitectonic features were compared with the corresponding coronal levels in a standard rat brain atlas (Paxinos and Watson, The Rat Brain in Stereotaxic Coordinates; atlas interval 0.36 mm; right) to accurately define the boundaries between the BLA (green) and CeA (red). Scale bar: 500 μm. Solid and dashed lines are used only for visual guidance and do not indicate different experimental conditions.

Toluidine blue (Nissl)-stained sections were used for cytoarchitectonic delineation and volumetric analysis of the amygdala. Briefly, free-floating coronal sections were mounted onto gelatin-coated slides, air-dried, and stained with toluidine blue according to standard protocols. After differentiation, dehydration through graded ethanol, and clearing in xylene, sections were coverslipped with a neutral mounting medium. Cytoarchitectonic delineation and stereological volume estimation of the amygdala are illustrated in Figure 2.

The boundaries of the basolateral amygdala (BLA) and central amygdala (CeA) were delineated based on a standard rat brain atlas (Paxinos and Watson, The Rat Brain in Stereotaxic Coordinates), together with cytoarchitectonic criteria observed in Nissl-stained sections. Specifically, distinct neuronal cell bands, regional differences in neuronal density, and laminar organization were used to reliably distinguish amygdala subregions across serial sections. Delineation was performed consistently across animals to ensure anatomical comparability.

Amygdala volume estimation was performed using the Cavalieri principle. At low magnification (2.5×), a systematic point grid was superimposed onto each Nissl-stained section using stereological software (Carl Zeiss, Oberkochen, Germany). Grid points falling within the contours of the entire amygdala and its subregions (BLA and CeA) were counted.

The volume (*V*) was calculated using the formula:

$$V\;=\;t\;\times \;a\left(p\right)\;\times \;\Sigma P$$

where *t* represents the distance between sampled sections (0.6 mm), *a*(*p*) is the area associated with each grid point (0.02 mm^2^), and *ΣP* is the total number of grid points counted for each structure per animal [49].

### 2.6. Immunohistochemistry

Free-floating coronal brain sections were processed for GFAP immunohistochemistry using standard protocols. After permeabilization and blocking, sections were incubated with a rabbit anti-GFAP primary antibody (Abcam, Cambridge, UK; ab7260; 1:1000), followed by biotinylated secondary antibody and HRP–streptavidin amplification. Immunoreactivity was visualized using diaminobenzidine (DAB) as the chromogen. Sections were mounted, dehydrated, and coverslipped for subsequent stereological analysis. Detailed procedures are provided in the Supplementary Materials and Methods.

### 2.7. Stereological Analysis

The number of GFAP^+^ astrocytes in the basolateral amygdala (BLA) and central amygdala (CeA) were estimated using the optical fractionator method. Amygdala subregions were delineated at low magnification (2.5×) based on Nissl-defined cytoarchitectonic criteria, with reference to a standard rat brain atlas, using a ZEISS stereology system (Carl Zeiss, Oberkochen, Germany).

Sampling was performed using a systematic random sampling strategy to ensure that each counting site had an equal probability of being selected. Stereological counting was carried out using optical dissector frames that were systematically and randomly distributed across each region of interest with an area sampling fraction (*asf*) of 15%.

**Figure 3:**
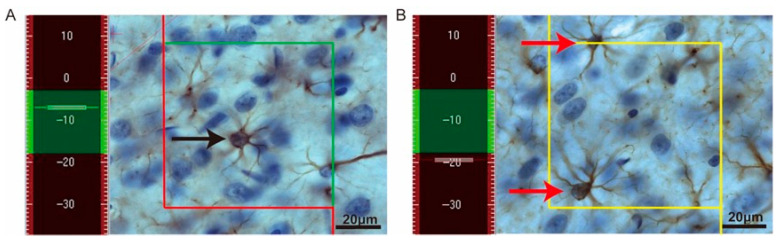
Unbiased counting rules for stereological quantification of GFAP^+^ astrocytes. (**A**) Representative high-magnification image illustrating the application of an unbiased counting frame for stereological analysis of GFAP^+^ astrocytes. The green line indicates the inclusion boundary, whereas the red lines and their extensions indicate the exclusion boundaries. A GFAP^+^ astrocyte nucleus (black arrow) was included in the count because it was located entirely within the counting frame or only intersected the inclusion boundary. (**B**) Representative image illustrating astrocytes excluded from stereological counting. Astrocyte nuclei (red arrows) were not counted because they intersected the exclusion boundaries. The yellow frame indicates regions outside the defined counting thickness (dissector height), and cells within this region were excluded from the counting. Scale bar: 20 μm.

Immunohistochemically stained sections were used for analysis. A guard zone of 3 μm was applied at the upper surface of each section to avoid lost caps, and GFAP^+^ astrocyte nuclei were counted within the subsequent 15-μm dissector height. Only cells that came into focus within the dissector height and met the unbiased counting frame criteria were included, i.e., cells located entirely within the counting frame or intersecting only the inclusion boundary, as illustrated in Figure 3.

The total number of astrocytes (*N*) was estimated using the formula:

$$N\;=\;\Sigma Q^{-}\;\times \;\frac{1}{ssf}\;\times \;\frac{1}{asf}\;\times \;\frac{1}{hsf}$$

where *ΣQ*^−^ represents the total number of counted GFAP^+^ cells, *ssf* is the section sampling fraction, *asf* is the area sampling fraction, and *hsf* is the height sampling fraction [50].

### 2.8. Immunofluorescence

For immunofluorescence analyses, free-floating sections were processed using standard double-labeling protocols. GFAP/BrdU immunofluorescence was performed to assess astrocyte proliferation, and GFAP/PSD95 double immunofluorescence was used to evaluate astrocyte–synapse associations. Primary antibodies included anti-GFAP (mouse monoclonal, Santa Cruz Biotechnology, Dallas, TX, USA; sc-33673; 1:500), anti-BrdU (rat monoclonal, Abcam, Cambrige, UK; ab6326; 1:1000), and anti-PSD95 (rabbit monoclonal, Cell Signaling Technology, Danvers, MA, USA; #3450; 1:500), followed by species-appropriate fluorescent secondary antibodies. Sections were mounted with antifade medium and imaged using a laser scanning confocal microscope (Andor, Belfast, UK). Detailed staining procedures, antibody dilutions, and imaging parameters are provided in the Supplementary Materials and Methods.

### 2.9. Statistics

Data were presented as mean ± SD and analyzed using SPSS Statistics 29.0 (IBM Corp, Armonk, NY, USA). Normality and homogeneity of variances were assessed using the Shapiro–Wilk and Levene’s tests, respectively. For longitudinal measures such as sucrose preference and body weight, repeated-measures ANOVA was performed. For multiple-group comparisons, one-way ANOVA followed by LSD post hoc tests was used when variances were homogeneous, whereas Brown–Forsythe analysis with Tamhane’s T2 post hoc tests was applied in cases of heteroscedasticity. When data did not meet the assumptions of normality, non-parametric tests were employed, including the Kruskal–Wallis test for comparisons among multiple groups and the Mann–Whitney U test for pairwise comparisons (e.g., behavioral parameters from the elevated plus maze and open field tests). Coefficients of variation (CV) and error (CE) were calculated, and power analysis was conducted to ensure sufficient statistical robustness. Sample sizes were determined based on prior studies, and statistical significance was set at *p* < 0.05.

### 2.10. Additional Materials and Methods

Detailed descriptions of the methods, materials, and additional statistical procedures are provided in the Supplementary Materials and Methods and Supplementary Table S1. Full statistical outputs for all results are reported in Supplementary Tables S2–S5.

## 3. Results

### 3.1. Running Exercise Selectively Alleviates CUS-Induced Anhedonia-like Behaviors in Rats

**Figure 4:**
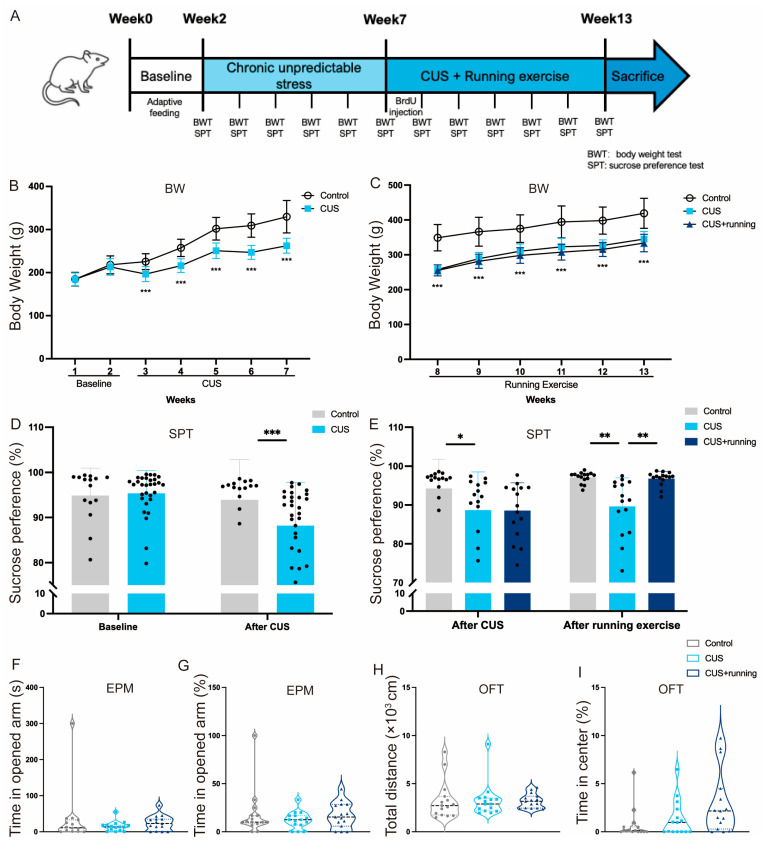
Effects of running exercise on anhedonia-like behaviors in CUS rats. (**A**) Experimental timeline. (**B**) Body weight of the control group (*n* = 15) and CUS group (*n* = 30) during the first 7 weeks. (**C**) Body weight of the control (*n* = 15), CUS (*n* = 15), and CUS+running (*n* = 15) groups during the running intervention period (weeks 8–14). (**D**) Sucrose preference in the control (*n* = 15) and CUS (*n* = 30) groups at baseline and after 5 weeks of CUS exposure. (**E**) Sucrose preference in the control (*n* = 15), CUS (*n* = 15), and CUS+running (*n* = 15) groups after the running intervention. (**F**,**G**) Time spent in the open arm of the elevated plus maze test in the control (*n* = 15), CUS (*n* = 15), and CUS+running (*n* = 15) groups. (**H**,**I**) Total distance and time spent in the center of the open field test for each group (*n* = 15 per group). Behavioral assessments in the OFT and EPM were conducted after the running intervention. For panels (**F**–**I**), violin plots display the distribution of the data, with individual data points representing single animals overlaid. Data are presented as mean ± SD. Significance levels: * *p* < 0.05, ** *p* < 0.01, *** *p* < 0.001. CUS, chronic unpredictable stress; BW, body weight; SPT, sucrose preference test; EPM, elevated plus maze test; OFT, open field test.

The experimental timeline is shown in Figure 4A. Behavioral tests were performed to evaluate the effects of running exercise on CUS-induced behavioral alterations. At the baseline, no significant differences were observed between the control and CUS groups in either body weight or sucrose preference (Figure 4B,D), indicating comparable initial conditions. After five weeks of CUS exposure, rats in the CUS group exhibited a significant reduction in body weight gain and sucrose preference compared with the controls (Figure 4B,D), consistent with stress-induced metabolic changes and the emergence of an anhedonia-like behavioral change. During the subsequent six-week intervention period, both the CUS and CUS+running groups maintained lower body weight than the controls (Figure 4C), indicating that running exercise did not normalize stress-associated alterations in body weight. Although running exercise improved anhedonia-like behavior, it did not normalize stress-induced body weight gain, indicating a dissociation between behavioral and physiological outcomes. In contrast, sucrose preference was significantly higher in the CUS+running group than in the CUS group at the end of the intervention period (Figure 4E), indicating a selective improvement in anhedonia-like behavior. No significant differences were observed among groups in elevated plus maze or open field test measures following the running intervention (Figure 4F–I), suggesting that anxiety-related behaviors were not significantly affected under the present experimental conditions. Together, these results indicate that within the behavioral domains assessed in the present study, CUS induced a prominent anhedonia-like behavioral deficit, and that running exercise selectively ameliorated this reward-related behavioral alteration without producing detectable changes in anxiety-related behavioral domains assessed by EPM and OFT.

### 3.2. Running Exercise Restores CeA Volume in CUS Rats

**Figure 5:**
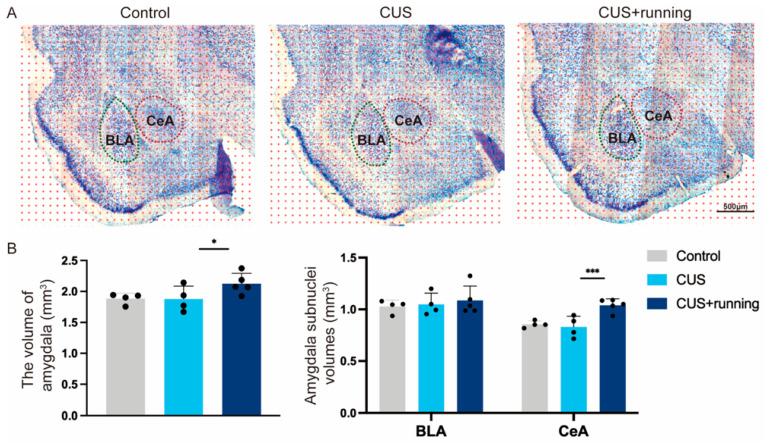
Running exercise restores CeA volume in CUS rats. (**A**) Representative Nissl-stained coronal sections of the amygdala from the control, CUS, and CUS+running groups. Stereological point-counting grids were superimposed for volumetric estimation. BLA is outlined in green and CeA is outlined in red, based on cytoarchitectonic criteria. Scale bars: 500 μm. (**B**) Quantification of the volumes of the BLA and CeA in the control (*n* = 4), CUS (*n* = 4), and CUS+running (*n* = 5) groups. Data are presented as mean ± SD. Significance levels: * *p* < 0.05, *** *p* < 0.001. CUS, chronic unpredictable stress; BLA, basolateral amygdala; CeA, central amygdala.

To investigate structural correlates of running exercise in CUS rats, amygdalar volume was assessed using stereological point counting on Nissl-stained coronal sections. As illustrated in Figure 5A, representative point-counting grids superimposed on the amygdala are shown for the control, CUS, and CUS+running groups. BLA and CeA were delineated based on cytoarchitectonic criteria, and volumetric estimates were obtained using systematic random sampling across serial sections. Compared with the control group, Compared with the control group, neither BLA nor CeA volume showed a statistically significant reduction after CUS exposure (Figure 5B). However, running exercise significantly increased CeA volume in CUS rats (Figure 5B). These results indicate that running exercise selectively modulates CeA volume under chronic stress conditions, whereas CUS alone does not produce a statistically significant reduction in CeA volume. No significant volumetric alterations were observed in the BLA across experimental groups.

### 3.3. Running Exercise Increases GFAP^+^ Astrocyte Numbers in the BLA and CeA of CUS Rats

**Figure 6:**
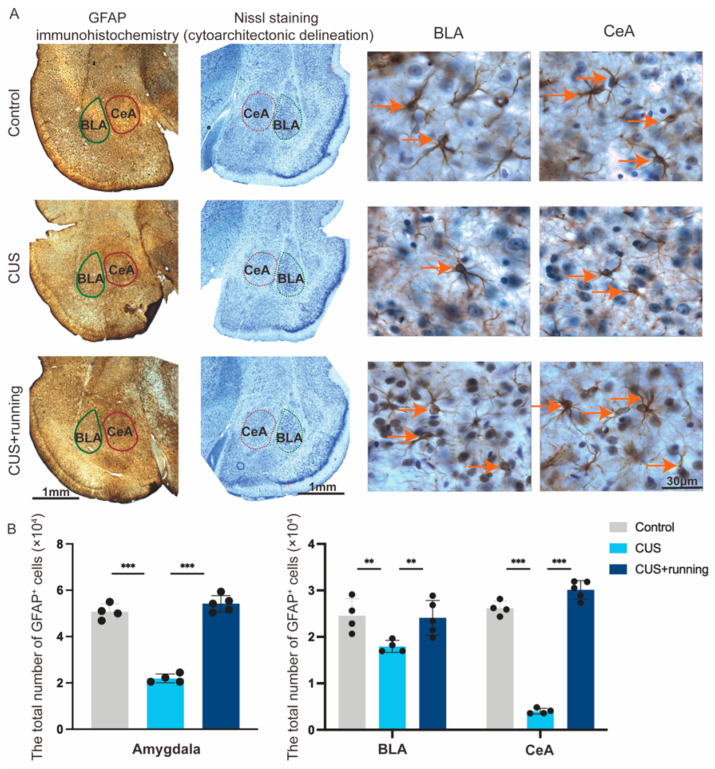
Running increases GFAP^+^ astrocyte numbers in the BLA and CeA of CUS rats. (**A**) Representative images of GFAP immunohistochemistry and corresponding Nissl staining in the amygdala from the control, CUS, and CUS+running groups. GFAP-stained sections and Nissl-stained sections were obtained from adjacent serial brain sections. Cytoarchitectonic boundaries of the BLA (green) and CeA (red) were identified based on neuronal cell bands visible in the Nissl-stained sections and subsequently transferred to the corresponding GFAP-stained sections. Solid and dashed lines indicate the boundaries of the BLA and CeA, respectively, as identified based on cytoarchitectonic criteria. Higher-magnification images of GFAP^+^ astrocytes in the BLA and CeA are shown on the right; arrows indicate representative GFAP^+^ astrocytes. Scale bar: 1 mm (low-magnification images) and 30 μm (high-magnification images). (**B**) Stereological estimates of total GFAP^+^ astrocyte numbers in the entire amygdala (left) and in the BLA and CeA (right) in the control (*n* = 4), CUS (*n* = 4), and CUS+running (*n* = 5) groups. Data are presented as mean ± SD. Significance levels: ** *p* < 0.01, *** *p* < 0.001. CUS, chronic unpredictable stress; GFAP, glial fibrillary acidic protein; BLA, basolateral amygdala; CeA, central amygdala.

To quantify astrocytes in amygdala subregions, GFAP-positive (GFAP^+^) astrocytes were assessed using immunohistochemistry combined with stereological analysis (Figure 6A). Representative GFAP staining is shown in Figure 6A, and stereological estimates of GFAP^+^ astrocyte numbers in the BLA and CeA are summarized in Figure 6B and Table 1. CUS significantly reduced the number of GFAP^+^ astrocytes in both the BLA and CeA compared with the controls (Figure 6B). Running exercise significantly increased the GFAP^+^ astrocyte numbers in both subregions relative to the CUS group (Figure 6B), indicating a restoration toward control levels. Sampling reliability was supported by stereological precision metrics, including the observed coefficient of error (OCE) and observed coefficient of variation (OCV), as reported in Table 1. These results suggest that chronic stress reduces astrocyte abundance in the amygdala and that running exercise promotes astrocyte population recovery in both BLA and CeA.

### 3.4. Running Exercise Restores Stress-Reduced Astrocytic Morphological Complexity in the BLA and CeA

**Figure 7:**
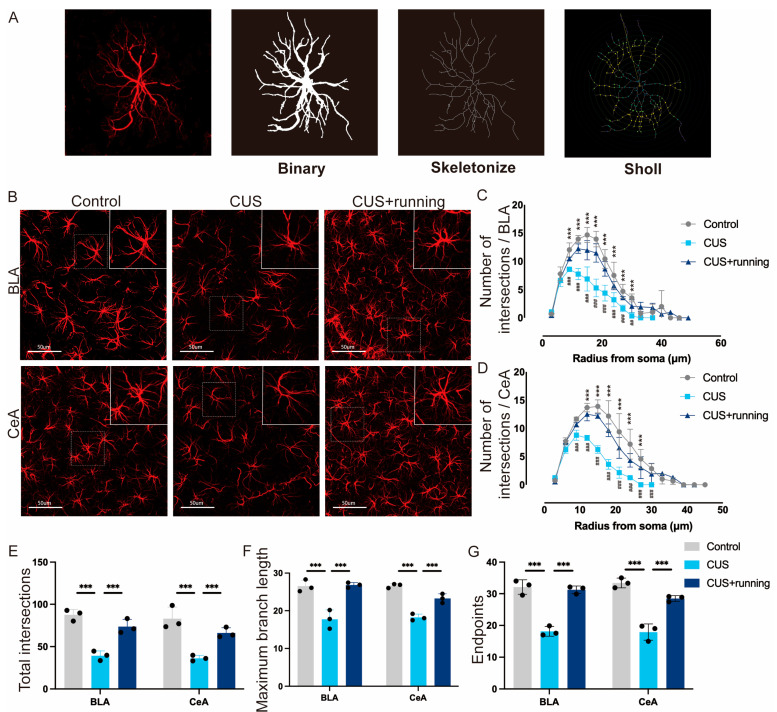
Running exercise enhances astrocyte structural complexity in the BLA and CeA of CUS rats. (**A**) Schematic illustration of astrocyte morphological quantification. Process length and endpoints were measured using the Analyze Skeleton plugin in ImageJ (version 1.54p, National Institutes of Health, Bethesda, MD, USA), and Sholl intersections were quantified using the Sholl Analysis plugin. (**B**) Representative images of GFAP (red) immunofluorescence staining in the BLA and CeA from the control, CUS, and CUS+running groups. Images were acquired using a 60× oil-immersion objective. Scale bar: 50 μm. (**C**,**D**) Quantification of astrocyte branching complexity by counting Sholl intersections from the soma in the BLA (**C**) and CeA (**D**) in the three groups (*n* = 3 animals per group; 18 astrocytes analyzed per animal). (**E**–**G**) Total intersections, maximum branch length, and number of endpoints for each astrocyte in the amygdalar subregions in the three groups (*n* = 3 animals per group; 18 astrocytes analyzed per animal). Data are presented as mean ± SD. Significance levels: *** *p* < 0.001; ## *p* < 0.01, ### *p* < 0.001 vs. CUS group. Statistical analyses were performed using the animal as the unit of analysis, with astrocytic measurements averaged within each animal. CUS, chronic unpredictable stress; GFAP, glial fibrillary acidic protein; BLA, basolateral amygdala; CeA, central amygdala.

To assess astrocyte morphology, GFAP immunofluorescence and three-dimensional imaging were performed, followed by skeleton and Sholl analyses. Representative images, skeleton reconstructions, and Sholl profiles from the BLA and CeA are shown in Figure 7A,B. CUS resulted in reduced astrocytic branching complexity, reflected by decreased Sholl intersections in both subregions (Figure 7C,D). Following running exercise, astrocytes in CUS rats exhibited significantly increased branching complexity, reaching levels comparable to the controls (Figure 7C,D). In addition, CUS reduced the number of process endpoints and maximum branch length (Figure 7E–G). These parameters were significantly restored by running exercise (Figure 7E–G; sampling details in Table 2). Collectively, these results indicate that chronic stress compromises astrocyte structural integrity in the BLA and CeA, whereas running exercise reverses these morphological deficits.

### 3.5. Running Exercise Increases BrdU^+^/GFAP^+^ Astrocytes in the BLA and CeA of CUS Rats

**Figure 8:**
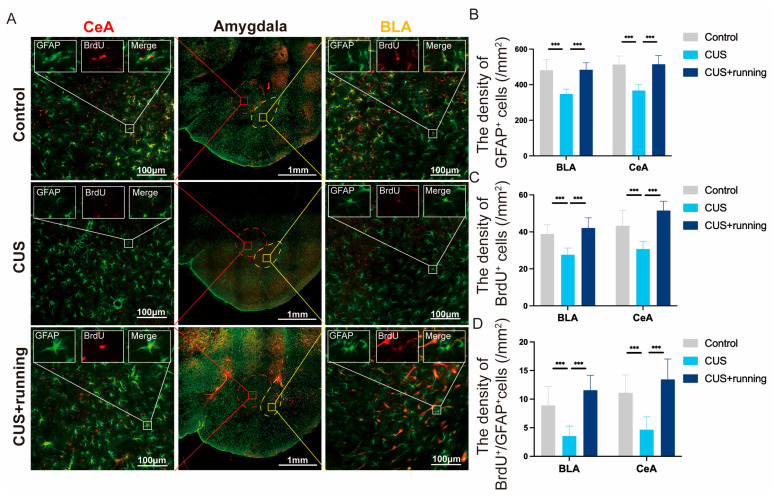
Running exercise increases BrdU^+^/GFAP^+^ astrocytes in the BLA and CeA of CUS rats. (**A**) Representative images of immunofluorescence staining for GFAP (green) and BrdU (red) in the BLA and CeA from the control, CUS, and CUS+running group. Scale bar: 50 μm. (**B**) The density of GFAP^+^ cells in the BLA and CeA in the three groups (*n* = 3 animals per group). (**C**) The density of BrdU^+^ cells in the BLA and CeA in the three groups (*n* = 3 animals per group). (**D**) The density of BrdU^+^/GFAP^+^ cells in the BLA and CeA in the three groups (*n* = 3 animals per group). Data are presented as mean ± SD. Significance levels: *** *p* < 0.001. CUS, chronic unpredictable stress; GFAP, glial fibrillary acidic protein; BrdU, bromodeoxyuridine; BLA, basolateral amygdala; CeA, central amygdala.

To determine whether changes in astrocyte abundance were associated with altered astrocyte proliferation, BrdU and GFAP double immunofluorescence was performed (Figure 8A). CUS significantly reduced the densities of GFAP^+^ cells and BrdU^+^ cells in both the BLA and CeA compared with the controls (Figure 8B,C). Running exercise restored the densities of both GFAP^+^ and BrdU^+^ cells (Figure 8B,C). Consistently, the density of BrdU^+^/GFAP^+^ cells (BrdU-labeled astrocytes) was significantly reduced in the CUS group and increased following running exercise (Figure 8D; sampling details in Table 3). These findings suggest that chronic stress suppresses astrocyte proliferation/renewal in the amygdala, whereas running exercise promotes the recovery of BrdU-labeled astrocytes in both subregions.

### 3.6. Running Exercise Increases Astrocyte-Associated PSD95^+^ Puncta in the Amygdala of CUS Rats

**Figure 9:**
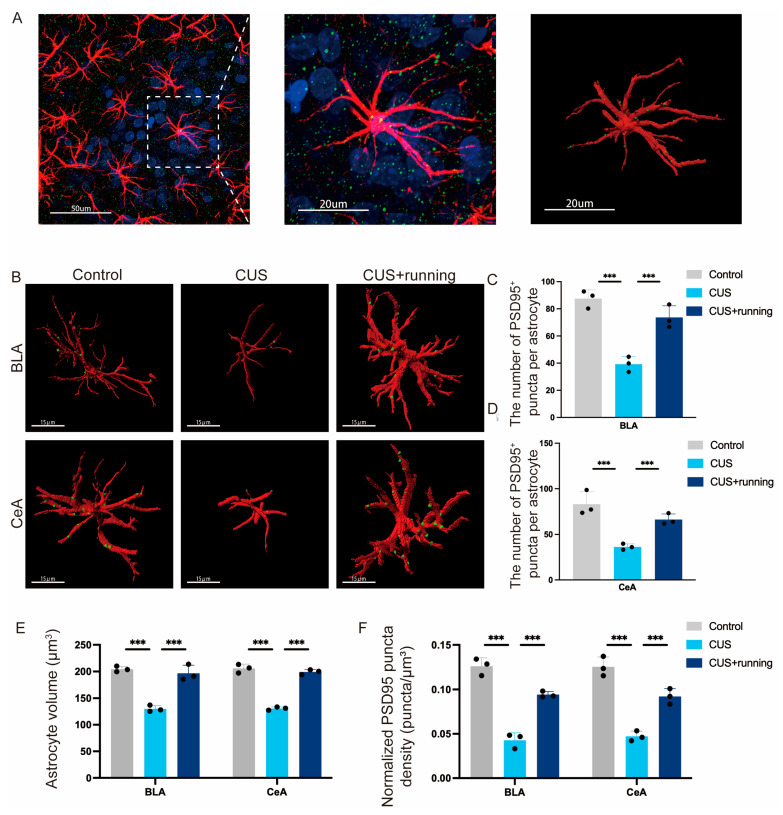
Running exercise increases astrocyte-associated PSD95^+^ puncta in the BLA and CeA. (**A**) Schematic illustration of the workflow for quantifying astrocyte-associated PSD95^+^ puncta using Imaris-based 3D reconstruction. (**B**) Representative fluorescence images of GFAP (red) and PSD95 (green) in the BLA and CeA from the control, CUS, and CUS+running groups. Three-dimensional (3D) reconstruction of astrocyte-associated PSD95 puncta was generated from confocal Z-stack images, preserving the original spatial dimensions of astrocytic processes and synaptic structures. Brightness and contrast were adjusted uniformly across all images for visualization purposes only. Scale bar: 15 μm. (**C**,**D**) Number of PSD95^+^ puncta associated with GFAP^+^ astrocytes in the BLA (**C**) and CeA (**D**). (**E**) Quantification of astrocyte volume in the BLA and CeA. (**F**) Quantification of normalized PSD95^+^ puncta density per astrocyte in the BLA and CeA. Normalized PSD95^+^ puncta density was calculated as the number of astrocyte-associated PSD95^+^ puncta divided by astrocyte volume (puncta/μm^3^). Data are presented as mean ± SD (*n* = 3 animals per group). Significance levels: *** *p* < 0.001. CUS, chronic unpredictable stress; BLA, basolateral amygdala; CeA, central amygdala; GFAP, glial fibrillary acidic protein; PSD95, postsynaptic density protein 95.

To examine excitatory synaptic alterations associated with astrocytic remodeling, astrocyte-associated PSD95^+^ puncta were quantified from 3D-reconstructed images using Imaris software (version 10.1.0, Oxford Instruments, Abingdon, UK) (Figure 9A,B). In the BLA, CUS significantly reduced the number of PSD95^+^ puncta associated with GFAP^+^ astrocytes compared with the control group, whereas running exercise significantly increased this parameter relative to the CUS group (Figure 9C). A similar pattern was observed in the CeA, where CUS decreased the number of PSD95^+^ puncta associated with astrocytes, and running exercise partially restored this reduction (Figure 9D). Sampling data are summarized in Table 4. In parallel, astrocyte volume was significantly reduced by CUS and restored by running exercise in both amygdala subregions (Figure 9E; Table 5). Furthermore, normalized PSD95^+^ puncta density, calculated as astrocyte-associated PSD95^+^ puncta number divided by astrocyte volume, was also significantly decreased after CUS and significantly increased following running exercise in both the BLA and CeA (Figure 9F). These findings suggest that running exercise attenuates stress-induced astrocytic atrophy and restores astrocyte-associated excitatory synaptic structures in the amygdala.

## 4. Discussion

Although running exercise is widely recognized as a beneficial non-pharmacological intervention for stress-related mood disturbances, the cellular and circuit-level mechanisms underlying its effects remain incompletely understood. In the present study, we combined behavioral, structural, cellular, and synaptic analyses to examine how running exercise modulates chronic stress-related alterations in the amygdala. Importantly, our findings are interpreted within the specific behavioral domains assessed in this study. The main findings can be summarized as follows: (i) running exercise selectively improved sucrose preference in CUS rats without producing significant changes in anxiety-related behavioral measures; (ii) CUS was associated with a reduction in CeA volume, and running exercise reversed this stress-related structural alteration; (iii) chronic stress reduced astrocyte abundance, proliferation, and morphological complexity in both the BLA and CeA, whereas running exercise restored these astrocytic features toward control levels; (iv) running exercise increased astrocyte-associated PSD95^+^ puncta, suggesting recovery of astrocyte–excitatory synapse coupling in amygdala subregions. Together, these results support a model in which running exercise ameliorates stress-induced anhedonia-like behavior in parallel with astrocyte-centered structural plasticity and synaptic remodeling in emotion-related amygdala subregions.

### 4.1. Anhedonia-like Behavioral Improvement and Amygdala Structural Plasticity

At the behavioral level, chronic unpredictable stress (CUS) produced a marked reduction in sucrose preference, a widely accepted index of anhedonia-like behavior [51,52]. Running exercise significantly restored sucrose preference in CUS rats, whereas elevated plus maze (EPM) and open field test (OFT) measures remained unchanged (Figure 4). These findings indicate that within the behavioral domains assessed in the present study, running exercise selectively improved reward-related behavior rather than anxiety-like behavior. Accordingly, our data do not support a generalized improvement across multiple affective dimensions, but instead point to a preferential modulation of anhedonia-related processes.

In recent years, anhedonia and behavioral despair have increasingly been recognized as related yet partially dissociable affective dimensions, supported by neural circuits that overlap but are not identical. For example, despair-like behaviors assessed by paradigms such as the forced swim test and tail suspension test are commonly interpreted as passive stress-coping responses and are highly sensitive to monoaminergic mechanisms, particularly serotonergic and noradrenergic pathways [53,54]. In contrast, reward-related behaviors, including sucrose preference, rely more heavily on limbic reward circuitry, in which the amygdala serves as an important integrative node [55,56]. In this context, the selective improvement in sucrose preference observed in the present study is consistent with the amygdala-centered mechanistic framework of our investigation, given the established role of the amygdala in reward-related valence processing and incentive integration [57,58]. Therefore, the behavioral effects reported here are more appropriately interpreted as the recovery of reward-related behavioral function, rather than a generalized improvement in a global “depression-like” phenotype.

Our stereological analysis further revealed that BLA volume remained relatively preserved under chronic stress (Figure 5). Notably, running exercise significantly increased CeA volume in CUS rats, suggesting that exercise may promote structural resilience or activity-dependent plasticity in this amygdala subregion. Amygdala volume alterations have been reported as markers of dysfunction in emotion-related circuits. High-resolution MRI studies have shown reduced amygdala volumes in patients with mood disorders or stress-related affective conditions compared with healthy controls [59], and bilateral amygdala reductions have been documented in depressed individuals, with untreated patients exhibiting a negative association between amygdala volume and the number of depressive episodes [60]. Meta-analytic evidence further indicates that medication status can influence amygdala volume, with volume loss often reported in untreated patients and volume increases observed in medicated patients [61]. In this context, our data suggest that chronic stress may preferentially impact specific amygdala subregions, and that running exercise can counteract stress-related structural vulnerability in the CeA.

While adaptive changes in amygdala subregions in response to exercise remain incompletely characterized, previous studies have reported that exercise can modulate anxiety-related behaviors through amygdala circuits, including the regulation of postsynaptic inhibitory proteins in the BLA and alterations in amygdala subregional connectivity [62,63]. Notably, however, such anxiety-modulatory effects were not observed in the present CUS paradigm, as running exercise failed to produce significant changes in EPM or OFT measures.

Although CUS failed to induce significant CeA shrinkage, it may have induced subthreshold functional or molecular disturbances (e.g., altered glucocorticoid signaling or reduced synaptic plasticity) that are not captured by volumetric analysis alone [64,65]. The selective increase in CeA volume observed exclusively in the CUS+running group may be attributed to the robust gliogenic effects of exercise. Our stereological data indicate that running increased astrocyte number and complexity specifically in stressed rats. Given the established astrocyte-promoting and gliogenic effects of exercise, together with evidence that reactive astrocytes undergo hypertrophy and structural remodeling, the selective volumetric expansion of the CeA may reflect, at least in part, exercise-associated astrocytic plasticity [66,67]. Rather than necessarily indicating pathology, this astrocytic hypertrophy may reflect adaptive astrocyte remodeling that helps preserve homeostasis, meet increased local metabolic demand, and strengthen astrocyte–synapse interactions [67,68], consistent with our observed increase in astrocyte-associated PSD95 puncta. The significant volumetric increase in the CUS+running group may indicate exercise-associated adaptive remodeling of the CeA, consistent with a resilience-related phenotype under ongoing stress [69,70]. This interpretation accords with the broader view that effective psychiatric interventions may act not only by reversing pathological deficits, but also by enhancing neuroplasticity and adaptive capacity [71].

It is noteworthy that despite robust changes in astrocyte number and morphology, BLA volume remained relatively unchanged across experimental groups. This apparent dissociation suggests that cellular-level alterations do not necessarily translate into detectable changes at the regional volumetric level [72]. The BLA is a neuron-rich structure in which gross volume is influenced by multiple tissue components, including neuronal somata, dendritic and axonal processes, synaptic neuropil, and extracellular space, rather than by glial cell number alone [73,74]. Under chronic stress conditions, astrocytic loss or remodeling may coexist with compensatory neuronal structural adaptations, such as dendritic reorganization, which could contribute to the preservation of overall regional volume [75,76]. Similar dissociations between microstructural remodeling and gross volumetric measures have been reported in stress-related and affective brain regions [72]. Therefore, unchanged BLA volume should not be interpreted as an absence of structural plasticity but rather as a reflection of complex multi-scale remodeling processes within this subregion.

This discrepancy may reflect differences in stress paradigms, exercise protocols, behavioral assay sensitivity, or the recruitment of distinct amygdala subcircuits [70,77,78]. Importantly, the present findings suggest that under chronic unpredictable stress, running exercise may preferentially influence reward-related and anhedonia-associated processes rather than anxiety-related domains. In this context, the selective restoration of CeA volume and astrocyte-centered structural plasticity may reflect circuit-specific adaptations within the amygdala. Given emerging evidence that the central amygdala contributes not only to anxiety-related responses but also to appetitive and reward-related motivation, these changes may be more closely related to reward- and anhedonia-associated processes than to anxiety regulation [79,80,81]. Further studies will be needed to determine whether different exercise regimens, behavioral paradigms, or region-specific circuit mechanisms account for the limited effects of running exercise on anxiety-like behavior in the present model.

### 4.2. Restoration of Astrocyte Number and Morphology

A central finding of this study is that astrocytes in the BLA and CeA are sensitive to chronic stress and responsive to running exercise. We observed that CUS reduced astrocyte numbers in both subregions, whereas running exercise restored astrocyte abundance to control-like levels (Figure 6). Astrocytes in distinct amygdala subregions play critical roles in regulating local circuit activity and emotional behaviors. For instance, astrocytes in the BLA can modulate adjacent glutamatergic neurons through D-serine signaling, thereby supporting neuronal activity and appropriate risk assessment behaviors [82]. Our results extend accumulating evidence supporting an “astrocyte hypothesis” of stress-related behavioral dysfunction by demonstrating stress-associated astrocyte loss and exercise-related astrocyte recovery specifically within amygdala subregions.

In line with a broader neuroprotective role of exercise on astrocytes, a recent Alzheimer’s disease study reported that exercise can restore both astrocyte abundance and protective transcriptomic features of a specialized neurovascular-associated astrocyte subtype [83]. Although disease contexts differ, these findings support the possibility that exercise engages conserved astrocyte-centered repair programs. Importantly, the recovery of astrocyte number in our study was accompanied by the restoration of astrocytic morphology. Astrocyte function is critically dependent on morphological complexity [84]. We found that CUS induced marked astrocytic simplification, while running exercise significantly increased structural complexity (Figure 7). Such morphological atrophy is often associated with reduced synaptic coverage and impaired neurotransmitter clearance [35,85]. For example, perisynaptic astrocytic processes (PAPs) have been identified as key microstructures that sense and regulate synaptic environments, and PAPs can represent early sites of disruption in disease settings [86]. Thus, the stress-induced astrocytic atrophy observed in our model may reflect impaired perisynaptic engagement, whereas exercise-related morphological restoration may indicate improved capacity for synaptic support, metabolic coupling, and homeostatic regulation. Together, these findings suggest that running exercise improves not only astrocyte abundance but also astrocyte structural features linked to functional quality.

### 4.3. Contribution of BrdU-Labeled Astrocytes

Our study further showed that running exercise increased BrdU^+^/GFAP^+^ cells in both the BLA and CeA of CUS rats (Figure 8). Because BrdU incorporation marks proliferating cells, we interpret BrdU^+^/GFAP^+^ cells as proliferating or recently generated astrocytes, consistent with enhanced astrocyte proliferation and/or renewal. These data suggest that exercise-related astrocyte recovery may involve not only the preservation of existing astrocytes but also replenishment of the astrocyte pool. Early animal studies reported that running exercise can enhance astrocyte proliferation in the frontal cortex and striatum [87]. In addition, moderate running has been shown to improve astrocytic coverage of microvessels, reduce inflammation, and restore PSD95 expression while improving cognitive performance [88], consistent with the coordinated regulation of glial, vascular, and synaptic microenvironments.

If newly proliferated astrocytes successfully mature and integrate into local networks, they may contribute to longer-term circuit stability. Several mechanisms may support this possibility. New or immature astrocytes can secrete synaptogenic molecules such as thrombospondins, promoting the formation of structurally normal but functionally developing synapses [89]. Astrocytes also regulate dendritic spine formation and NMDA receptor-dependent synaptic integration via D-serine signaling [90]. Moreover, the “tripartite synapse” framework positions astrocytes as active participants in synaptic communication that can sense neurotransmitters and modulate synaptic transmission and plasticity [91]. Beyond synaptogenesis, astrocytes maintain network stability through ion homeostasis, metabolic support, and neurovascular coupling [92,93]. Thus, exercise-associated increases in BrdU-labeled astrocytes may contribute to the restoration of circuit function through both synaptic and homeostatic mechanisms.

### 4.4. Astrocyte–Excitatory Synapse Interactions

A further strength of this study is the linkage between astrocytic alterations and excitatory synaptic structure. We found that CUS reduced the number of astrocyte-associated PSD95^+^ puncta, whereas running exercise restored these astrocyte–synapse associations (Figure 9). Given that amygdala circuits—including glutamatergic projections related to BLA–CeA information flow—are critical for affective behaviors, stress-related changes in excitatory synaptic plasticity within these pathways likely contribute to stress-related anhedonia-like behavioral changes [94]. Our findings therefore provide mechanistic insight into how exercise may influence synaptic microenvironments in emotion-related circuits.

Astrocytes enwrap synapses via perisynaptic processes, forming tripartite synapses and regulating synaptic transmission and plasticity [95]. PSD95 is a core scaffolding protein of glutamatergic postsynaptic densities and is essential for synaptic stability, receptor anchoring, and signal transduction [96]. Reductions in PSD95 have been reported in both the hippocampus [97] and prefrontal cortex [98] of depressive rodent models. A recent study in *Cell* further suggested that astrocytes may coordinate groups of synapses as functional clusters rather than acting solely at individual synapses [99], highlighting astrocytic contributions to circuit-level computation.

In CUS rats, reduced astrocyte-associated PSD95^+^ puncta may reflect impaired astrocyte–synapse coupling that disrupts excitatory signaling homeostasis and contributes to behavioral dysfunctions and contributes to stress-related behavioral dysfunctions, particularly in the domain of reward-related processing [94]. Reduced astrocyte-associated PSD95^+^ puncta under CUS may partly reflect astrocytic atrophy rather than purely synapse-specific alterations [100]. Because astrocytic processes contribute to synaptic ensheathment, stress-induced reductions in astrocyte volume or morphological complexity could secondarily decrease the number of detectable astrocyte-associated synaptic contacts [38,101]. To address this possibility, PSD95 puncta counts were normalized to astrocyte volume. The normalized analysis indicated that the reduction in astrocyte-associated PSD95 puncta could not be explained solely by astrocytic atrophy, suggesting that stress may also disrupt astrocyte–synapse interactions [101]. Conversely, the restoration observed following running exercise likely reflects improvements in astrocyte-associated synaptic organization beyond structural preservation [85].

Running exercise restored astrocyte-associated PSD95^+^ puncta, consistent with a recovery of synaptic support. Exercise can activate astrocytic Ca^2+^ signaling, which may suppress excessive glutamate release via ATP/adenosine pathways and help prevent synaptic weakening [102]. Astrocytes also regulate synaptic strength through gliotransmitter release, enabling the modulation of synaptic activity and network plasticity [103]. Together with astrocytic roles in glutamate clearance, metabolic support, and ion buffering, these mechanisms suggest that running exercise may normalize astrocyte function and promote the restoration of excitatory connectivity in amygdala circuits, providing a plausible substrate for behavioral improvement [32,104]. Overall, our findings are consistent with emerging perspectives that astrocytes act as key regulators of excitatory network function in affect-related brain regions. Past studies have shown that patients with depression and animal models of depression exhibit abnormal neural circuitry and altered excitatory synaptic transmission in the BLA [105]. Meanwhile, astrocyte-released glutamate plays an important role in the formation of neurons and their synapses [106]. However, the observed recovery may reflect several mechanisms of synchronous changes, including astrocyte-mediated support of synaptic restoration, neuron-driven astrocytic remodeling, and reciprocal astrocyte–neuron interactions [107,108]. As the current analyses are conducted at a common endpoint, they cannot explain the chronological order or causal relationship of the recovery of astrocytes and neurons. Therefore, in the BLA and CeA of depression model mice, whether astrocyte-associated synaptic loss precedes or follows alterations in neuronal homeostasis, neural circuitry, and excitatory/inhibitory synaptic balance will be a key focus of our future investigations.

### 4.5. Limitations

Several limitations should be acknowledged. First, BrdU labeling identifies proliferating cells but does not by itself establish lineage origin or long-term functional integration; future studies incorporating astrocyte-lineage tracing and time-course analyses will be important. Second, the present findings are primarily correlational; astrocyte-specific manipulations will be necessary to test causality between astrocyte restoration, synaptic remodeling, and behavioral improvement. Third, the present study focused on the therapeutic effects of running exercise on depression. Nevertheless, the potential preventive effects of running also warrant further investigation. In future studies, we will design dedicated experiments to examine the preventive role of exercise in depression in parallel. Finally, only male rats were included, and potential sex-dependent effects should be examined in future work.

## 5. Conclusions

In conclusion, the present study demonstrates that chronic unpredictable stress induces pronounced astrocytic and synaptic alterations within amygdala subregions, accompanied by impaired reward-related behavior. Running exercise effectively reverses these stress-associated changes, including the restoration of astrocyte abundance, structural complexity, astrocyte-associated excitatory synaptic markers, and CeA volume, in parallel with an improvement of anhedonia-like behavior. These findings highlight astrocytes as central cellular mediators of exercise-induced resilience and support an astrocyte-centered structural plasticity mechanism within amygdala circuits that links physical activity to the regulation of stress-related reward dysfunction.

## Figures and Tables

**Table 1:** Sampling scheme and stereological precision for the quantification of GFAP^+^ astrocytes in the BLA and CeA.

	Control Group	CUS Group	CUS+Running Group
Number of sections sampled			
BLA	8–10	8–9	6–8
CeA	6–7	5–7	5–7
Section thickness (μm)			
BLA	22.56 ± 1.36	22.56 ± 1.36	24.35 ± 2.25
CeA	24.76 ± 5.92	24.76 ± 5.92	24.83 ± 2.32
Number of GFAP^+^ cells counted			
BLA	47 (16–64)	38 (12–54)	53 (18–84)
CeA	61 (22–103)	48 (23–68)	76 (26–112)
Amygdala	92 (49–148)	75 (40–134)	118 (40–191)
Total number of GFAP^+^ cells (×10^4^)			
BLA	2.46 ± 0.37	1.79 ± 0.13	2.41 ± 0.37
CeA	2.62 ± 0.16	0.40 ± 0.06	3.01 ± 0.20
Amygdala	5.07 ± 0.34	2.20 ± 0.19	5.42 ± 0.35
OCV (×10^−3^)			
BLA	125.74	135.55	174.72
CeA	123.64	166.93	131.39
Amygdala	71.55	90.97	114.43
OCE (×10^−3^)			
BLA	49.76	55.45	52.53
CeA	50.31	59.26	47.21
Amygdala	31.60	36.12	34.96
OCE^2^/OCV^2^ (×10^−3^)			
BLA	156.60	167.34	90.39
CeA	165.51	126.02	129.12
Amygdala	195.06	157.63	93.36

Table Note: The number of sampled sections is shown as a range. Section thickness and estimated total number of GFAP^+^ astrocytes are reported as mean ± SD. The number of counted GFAP^+^ cells are presented as mean (range). Abbreviations: BLA, basolateral amygdala; CeA, central amygdala; OCE, observed coefficient of error; OCV, observed coefficient of variation.

**Table 2:** Sampling scheme for astrocyte morphological parameters (total intersections, maximum branch length, and endpoints) in the BLA and CeA.

	Control Group	CUS Group	CUS+Running Group
Total intersections of astrocytes sampled			
BLA	87.32 (52–124)	40.04 (14–74)	73.70 (45–119)
CeA	84.16 (56–125)	36.29 (25–56)	66.19 (47–110)
Maximum branch length of astrocytes sampled (μm)			
BLA	26.57 (18.00–37.51)	17.75 (9.37–27.66)	26.83 (18.57–36.30)
CeA	26.70 (18.70–36.673)	18.38 (13.70–26.76)	23.26 (17.17–30.75)
Endpoints of astrocytes sampled			
BLA	31.90 (20–46)	18.29 (7–34)	31.15 (21–52)
CeA	33.23 (24–57)	18.19 (9–26)	28.48 (17–44)

Table note: Values are presented as mean (range). Abbreviations: BLA, basolateral amygdala; CeA, central amygdala; CUS, chronic unpredictable stress.

**Table 3:** Densities of GFAP^+^, BrdU^+^, and BrdU^+^/GFAP^+^ cells in the BLA and CeA.

	Control Group	CUS Group	CUS+Running Group
Density of GFAP^+^ cells (cells/mm^2^)			
BLA	481.67 ± 59.47	347.89 ± 27.33	483.67 ± 39.55
CV	0.12	0.08	0.08
CeA	513.22 ± 48.13	367.56 ± 32.18	515.22 ± 48.79
CV	0.09	0.09	0.09
Density of BrdU^+^ cells (cells/mm^2^)			
BLA	38.89 ± 4.99	27.67 ± 3.60	42.11 ± 5.49
CV	0.13	0.13	0.13
CeA	43.33 ± 8.26	30.78 ± 4.18	51.56 ± 5.03
CV	0.19	0.14	0.09
Density of BrdU^+^/GFAP^+^ cells (cells/mm^2^)			
BLA	8.89 ± 3.33	3.56 ± 1.74	11.56 ± 2.60
CV	0.38	0.49	0.22
CeA	11.11 ± 3.10	4.67 ± 2.23	13.44 ± 3.57
CV	0.28	0.48	0.27

Table note: Values are presented as mean ± SD. Abbreviations: BLA, basolateral amygdala; CeA, central amygdala; CV, coefficient of variation.

**Table 4:** PSD95^+^ puncta per astrocyte in the BLA and CeA.

	Control Group	CUS Group	CUS+Running Group
BLA	25.80 (9–70)	5.99 (1–16)	18.10 (6–35)
CeA	25.85 (11–75)	6.38 (3–13)	17.92 (6–38)

Table note: Values are presented as mean (range). Abbreviations: BLA, basolateral amygdala; CeA, central amygdala; CUS, chronic unpredictable stress; PSD95, postsynaptic density protein 95.

**Table 5:** Astrocyte volume in the BLA and CeA (μm^3^).

	Control Group	CUS Group	CUS+Running Group
BLA	204.44 (162–248)	130.15 (89–161)	196.70 (153–239)
CeA	205.70 (167–251)	130.70 (99–156)	199.22 (142–248)

Table note: Values are presented as mean (range). Abbreviations: BLA, basolateral amygdala; CeA, central amygdala; CUS, chronic unpredictable stress.

## Data Availability

The data presented in this study are available in the article and Supplementary Materials.

## Supplementary Materials

The following supporting information can be downloaded at: https://www.mdpi.com/article/10.3390/cells15080693/s1, Supplementary Materials and Methods: Detailed descriptions of animals and ethical approval, CUS intervention and experimental timeline, treadmill running protocol and BrdU administration, behavioral tests (SPT, EPM, OFT), perfusion and tissue preparation, toluidine blue staining and stereological volume estimation, immunohistochemistry/immunofluorescence, and stereological analysis; Table S1: Schedule of the CUS paradigm; Table S2: Full statistical outputs for Figure 1, Figure 2, Figure 3, Figure 4, Figure 5, Figure 6, Figure 7, Figure 8 and Figure 9; Table S3: Results of the elevated plus maze test; Table S4: Results of the open field test; Table S5: Stereological estimates of BLA and CeA volumes with measures of variability and sampling error.

## Abbreviations

BLA	Basolateral amygdala
BrdU	5-Bromo-2′-deoxyuridine (bromodeoxyuridine)
BW	Body weight
CeA	Central amygdala
CE	Coefficient of error
CUS	Chronic unpredictable stress
CV	Coefficient of variation
EPM	Elevated plus maze
GFAP	Glial fibrillary acidic protein
IL-6	Interleukin-6
IL-6R	Interleukin-6 receptor
Imaris	Imaris software
i.p.	Intraperitoneal
LSD	Least significant difference
MRI	Magnetic resonance imaging
NIH	National Institutes of Health
NMDA	N-Methyl-D-aspartate
OCE	Observed coefficient of error
OCV	Observed coefficient of variation
OFT	Open field test
PAPs	Perisynaptic astrocytic processes
PSD95	Postsynaptic density protein 95
SPSS	Statistical Package for the Social Sciences
SPT	Sucrose preference test
