# Supplementary material for: Running Exercise Promotes Astrocyte-Mediated Structural Plasticity in the Amygdalar BLA and CeA to Alleviate Anhedonia-like Behavior Alterations
Source: Cells. 2026 Apr 14;15(8):693. doi: 10.3390/cells15080693 (PMC13114546; doi:10.3390/cells15080693)
Supplement: Supplementary file 1 [file cells-15-00693-s001.zip › Supplementary Table S3.pdf]

Supplementary Table S3. Elevated plus maze test results.

	Control group (n=15)	CUS group (n=15)	CUS+running group (n=15)
Number of open-arm entries	1.27±0.88	1.07±0.70	1.60±1.18
Times spent in open arms (s)	33.58±74.92	14.97±13.95	24.13±20.87
Number of closed-arm entries	8.93±4.19	7.87±3.52	8.07±3.86
Time spent in closed arms (s)	188.74±68.24	207.01±48.17	150.73±58.03
Total arm entries	10.20±4.58	8.93±3.73	9.67±4.19
Percentage of open arm entries (%)	18.01±25.41	12.12±8.91	17.05±13.31
Percentage of time spent in open arms (%)	11.19±26.54	4.99±4.64	8.04±6.95

Table note: Data are presented as mean ± SD (n = 15 per group). Statistical comparisons were performed using one-way ANOVA.
