# Supplementary material for: Running Exercise Promotes Astrocyte-Mediated Structural Plasticity in the Amygdalar BLA and CeA to Alleviate Anhedonia-like Behavior Alterations
Source: Cells. 2026 Apr 14;15(8):693. doi: 10.3390/cells15080693 (PMC13114546; doi:10.3390/cells15080693)
Supplement: Supplementary file 1 [file cells-15-00693-s001.zip › Supplementary Table S2.pdf]

Supplementary Table S2:

**Fig.4**		
Fig.4B (Repeated measures ANOVA)	Body weight (baseline)	F (2,42) = 0.288; *p*=0.751
	Body weight (week 2-7)	F (2,42) =35.517; *p*<0.001
Fig.4C (Repeated measures ANOVA)	Body weight (week 8-13)	F (2,42) =18.730; *p*<0.001<br>Post-hoc: CUS *vs.* Control: *p*=0.000<br>CUS+running vs. CUS: *p*=0.562
Fig.4D (T test)	Sucrose preference (week=2)	t= -0.297; *p*= 0.768
	Sucrose preference (week=7)	t=2.183; *p*=0,035
Fig.4E (Mann-Whitney U test; Kruskal-Wallis test)	Sucrose preference (week=7)	U =362; *p*=0.001
	Sucrose preference (week=13)	H=19.20; df=2; *p*<0.001<br>Post-hoc: CUS *vs.* Control: *p*=0.035<br>CUS+running *vs.* CUS: *p*=0.004
Fig.4F (One-way ANOVA)	Elevated plus maze test / Time in opened arm (s)	F (2,42) =0.624; *p*=0.541
Fig.4G (Kruskal-Wallis test)	Elevated plus maze test / Time in opened arm (%)	H=1.55; df=2; *p*=0.462<br>Post-hoc: CUS *vs.* Control: *p*=0.99<br>CUS+running *vs.* CUS: *p*=0.612
Fig.4H (Kruskal-Wallis test)	Open field test / Total distance ($\times 10^3$ cm)	H=1.64; df=2; *p*=0.441<br>Post-hoc: CUS *vs.* Control: *p*>0.05<br>CUS+running *vs.* CUS: *p*>0.05
Fig.4I (Kruskal-Wallis test)	Open field test / Time in center (%)	H=7.33; df=2; *p*=0.026<br>Post-hoc: CUS vs. Control: *p*=0.99<br>CUS+running *vs.* CUS: *p*=0.236
**Fig.5**		
Fig.5C (one-way ANOVA)	Volume of BLA	F (2,10) =0.313; *p*=0.738<br>Post-hoc: CUS *vs.* Control: *p*=0.457<br>CUS+running *vs.* CUS: *p*=0.635
	Volume of CeA	F (2,10) =11.987; *p*=0.002<br>Post-hoc: CUS *vs.* Control: *p*=0.640<br>CUS+running *vs.* CUS: *p*=0.001
**Fig.6**		
Fig.6B (one-way ANOVA)	Number of GFAP+ cells /BLA	F (2,10) =5.572; *p*=0.024<br>Post-hoc: CUS *vs.* Control: *p*=0.015<br>CUS+running *vs.* CUS: *p*=0.016
	Number of GFAP+ cells /CeA	F (2,10) =350.1; *p*<0.001<br>Post-hoc: CUS *vs.* Control: *p*=0.000<br>CUS+running *vs.* CUS: *p*=0.000
**Fig.7**		

Fig.7C (Repeated measures ANOVA)	Number of intersections /BLA	F (30,64) =19.53; $p<0.001$
Fig.7D (Repeated measures ANOVA)	Number of intersections /CeA	F (28,55) =19.53; $p<0.001$
Fig.7E (one-way ANOVA)	Total intersections /BLA	F (2,6) =37.833; $p<0.001$<br>Post-hoc: CUS *vs.* Control: $p<0.001$<br>CUS+running *vs.* CUS: $p<0.001$
	Total intersections /CeA	F (2,6) =21.631; $p=0.001$<br>Post-hoc: CUS *vs.* Control: $p=0.001$<br>CUS+running *vs.* CUS: $p=0.001$
Fig.7F (one-way ANOVA)	Maximum branch length /BLA	F (2,6) =26.102; $p=0.002$<br>Post-hoc: CUS *vs.* Control: $p=0.001$<br>CUS+running *vs.* CUS: $p<0.001$
	Maximum branch length /CeA	F (2,6) =53.748; $p=0.001$<br>Post-hoc: CUS *vs.* Control: $p=0.001$<br>CUS+running *vs.* CUS: $p=0.001$
Fig.7G (one-way ANOVA)	Endpoints /BLA	F (2,6) =60.953; $p<0.001$<br>Post-hoc: CUS *vs.* Control: $p<0.001$<br>CUS+running *vs.* CUS: $p<0.001$
	Endpoints /CeA	F (2,6) =57.312; $p<0.001$<br>Post-hoc: CUS *vs.* Control: $p<0.001$<br>CUS+running *vs.* CUS: $p<0.001$
**Fig.8**		
fig.8B (one-way ANOVA)	Density of GFAP cells (/mm^2) /BLA	F (2,6) =46.73; $p<0.001$<br>Post-hoc: CUS *vs.* Control: $p<0.001$<br>CUS+running *vs.* CUS: $p<0.001$
	Density of GFAP cells (/mm^2) /CeA	F (2,6) =73.28; $p<0.001$<br>Post-hoc: CUS *vs.* Control: $p<0.001$<br>CUS+running *vs.* CUS: $p<0.001$
Fig.8C (one-way ANOVA)	Density of BrdU+ cells (/mm^2) /BLA	F (2,6) =51.94; $p<0.001$<br>Post-hoc: CUS *vs.* Control: $p<0.001$<br>CUS+running *vs.* CUS: $p<0.001$
	Density of BrdU+ cells (/mm^2) /CeA	F (2,6) =34.69; $p<0.001$<br>Post-hoc: CUS *vs.* Control: $p<0.001$<br>CUS+running *vs.* CUS: $p<0.001$
Fig.8D (one-way ANOVA)	Density of BrdU+/GFAP+ cells (/mm^2) /BLA	F (2,6) =56.00; $p<0.001$<br>Post-hoc: CUS *vs.* Control: $p=0.001$<br>CUS+running *vs.* CUS: $p<0.001$

	Density of BrdU+/GFAP+ cells (/mm^2) /CeA	F (2,6) =39.24; p<0.001<br>Post-hoc: CUS *vs.* Control: p<0.001<br>CUS+running *vs.* CUS: p<0.001
**Fig.9**		
Fig.9C (one-way ANOVA)	Number of PSD95$^+$ puncta per astrocyte /BLA	F (2,6) =54.91; p<0.001<br>Post-hoc: CUS *vs.* Control: p<0.001<br>CUS+running *vs.* CUS: p<0.001
Fig.9D (one-way ANOVA)	Number of PSD95$^+$ puncta per astrocyte /CeA	F (2,6) =157.67; p<0.001<br>Post-hoc: CUS *vs.* Control: p=0.000<br>CUS+running *vs.* CUS: p<0.001
