# Supplementary material for: Running Exercise Promotes Astrocyte-Mediated Structural Plasticity in the Amygdalar BLA and CeA to Alleviate Anhedonia-like Behavior Alterations
Source: Cells. 2026 Apr 14;15(8):693. doi: 10.3390/cells15080693 (PMC13114546; doi:10.3390/cells15080693)
Supplement: Supplementary file 1 [file cells-15-00693-s001.zip › Supplementary Materials and Methods.pdf]

Materials and Methods

Animals

Forty-five male Sprague–Dawley rats (4–6 weeks old, 150 ± 10 g) were obtained from Chongqing Medical University. Animals were housed in polycarbonate cages with autoclaved bedding under controlled conditions (22 ± 2 °C, 50 ± 5% humidity, 12 h/12 h light–dark cycle, lights on at 07:00). Food and water were provided ad libitum. After 7 days of acclimation (4–5 per cage), rats were randomly assigned to either a control group (CON, n = 15) or a chronic unpredictable stress group (CUS, n = 30). In this research, all rat experiments were performed according to the National Institutes of Health Guide for the Care and Use of Laboratory Animals (NIH Publication No. 85-23). All experiments were performed blindly and approved by the Ethics Committee of Chongqing Medical University (approval No. 2021067).

CUS intervention and experimental timeline

The CUS group was exposed to two different stressors per day for five weeks. Stressors were randomly selected from thermal challenge, light/dark disruption, noise, food or water deprivation, empty bottle, damp bedding, foot shock, restraint, tail pinch, and cage tilt. Control rats were maintained under standard housing conditions without stress exposure. Sucrose preference and body weight were measured weekly during the CUS period. After completion of CUS, CUS rats were randomly divided into a sedentary subgroup (the CUS group, n = 15) and a running exercise subgroup (the CUS+running group, n = 15). The control group remained sedentary throughout the experiment.

Exercise Protocol and BrdU Administration

Rats in the CUS+running group underwent six weeks of running exercise (20 min/day, 5 days/week) on a motorized six-lane treadmill. The speed was gradually increased from 10 m/min to 20 m/min during the first week and mained at 20 m/min thereafter, following an established protocol. The control and CUS groups did not participate in running exercise. During the first training week, the exercise rats received daily intraperitoneal injections of BrdU (20 mg/mL in saline; Sigma-Aldrich, #B5002) at 5 mL/kg for 7 consecutive days, as described a published method.

Sucrose Preference Test (SPT)

Anhedonia-like behavior was assessed using the sucrose preference test (SPT). The test was conducted weekly at 8:30 AM, immediately after body weight measurement. During each 24-hour session, rats had simultaneous access to two bottles: one containing plain water and the other containing 1% sucrose solution. The left–right position of the sucrose bottle was systematically alternated across cages to avoid side bias. Sucrose preference (%) was calculated as: (sucrose intake / total fluid intake) × 100%.

Body Weight

Body weight was measured weekly prior to the sucrose preference test.

Elevated Plus-Maze Test (EPM)

The elevated plus-maze test was used to assess anxiety-like behavior by measuring rodents' natural avoidance of open spaces versus their exploratory tendencies. Each rat was placed at the center of the maze and allowed to explore freely for 5 minutes. A video tracking system recorded the number of entries and the time spent in both the open and closed arms. Results were expressed as the percentage of time spent in open arms relative to the total time in all arms. An arm entry was defined as the rat's entire body (excluding the tail) fully entering an arm. The apparatus was cleaned with 75% ethanol between trials.

Open field test (OFT)

Locomotor activity and anxiety-like exploration were assessed in an open field arena (100 × 100 × 40 cm; opaque walls). Testing was conducted during the light phase under

dim illumination (approximately 20–30 lux). Each rat was placed gently in the center of the arena and allowed to explore freely for 5 min. Movements were recorded by an overhead camera and analyzed using an automated tracking system (e.g., ANY-maze or EthoVision XT). The arena floor was virtually divided into a center zone (50 × 50 cm) and a surrounding peripheral zone. Primary outcomes included total distance traveled, time spent in the center zone, number of center entries, and mean velocity. After each trial, the arena was cleaned with 75% ethanol and allowed to dry to remove olfactory cues.

Perfusion and Tissue Preparation

All subsequent procedures and analyses were performed under blinded conditions. Five rats were randomly selected from each group for stereological analysis. Animals were deeply anesthetized with 1% sodium pentobarbital (4 mL/kg, i.p.) and transcardially perfused with 4% paraformaldehyde (PFA) in 0.1 M phosphate-buffered saline (PBS, pH 7.4).

Following perfusion fixation, the brains were removed, and the meninges, cerebellum, and brainstem were carefully removed. One cerebral hemisphere (left or right) was randomly selected for subsequent analyses. The selected hemispheres were post-fixed in 4% PFA at 4 °C for at least 24 h and subsequently cryoprotected in graded sucrose solutions (10%–30% in PBS) at 4 °C until the tissue sank.

The hemispheres were then frozen at −60 °C and coronally sectioned at a thickness of 50 μm using a cryostat (Leica CM1860, Wetzlar, Germany). Serial sections were collected using a systematic random sampling strategy, with every sixth section selected for analysis (section sampling fraction, ssf = 1/6). Serial sections were collected using a systematic random sampling strategy (Figure 1). On average, approximately 12 sections spanning the amygdala were obtained from each hemisphere.

Prior to storage, sections were rinsed in PBS and ethanol and stored at −20 °C until further processing. For subsequent Nissl staining and GFAP immunohistochemistry, two series were randomly selected from the six serially collected section sets for each brain.

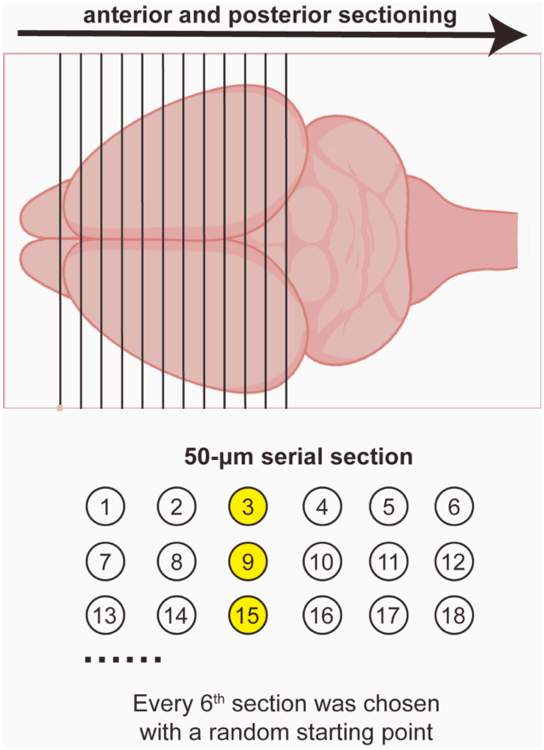

Figure 1. Systematic random sampling of serial coronal brain sections for stereological analysis. Serial coronal sections (50 μm thick) were collected along the anterior–posterior axis and systematically divided into six parallel series. With a random starting point, every sixth section was selected

for analysis (section sampling fraction, ssf = 1/6). For example, sections 3, 9, and 15 (highlighted in yellow) were chosen. This approach ensured systematic and unbiased sampling across the entire amygdala. Two series were randomly selected from the six section series for subsequent Nissl staining and GFAP immunohistochemistry.

Toluidine Blue (Nissl) Staining and Amygdala Volume Estimation

Toluidine blue (Nissl)-stained sections were used for cytoarchitectonic delineation and volumetric analysis of the amygdala. Briefly, free-floating coronal sections were mounted onto gelatin-coated slides, air-dried, and stained with toluidine blue according to standard protocols. After differentiation, dehydration through graded ethanol, and clearing in xylene, sections were coverslipped with a neutral mounting medium. Cytoarchitectonic delineation and stereological volume estimation of the amygdala are illustrated in Figure 2.

The boundaries of the basolateral amygdala (BLA) and central amygdala (CeA) were delineated based on a standard rat brain atlas (Paxinos and Watson, The Rat Brain in Stereotaxic Coordinates), together with cytoarchitectonic criteria observed in Nissl-stained sections. Specifically, distinct neuronal cell bands, regional differences in neuronal density, and laminar organization were used to reliably distinguish amygdala subregions across serial sections. Delineation was performed consistently across animals to ensure anatomical comparability.

Amygdala volume estimation was performed using the Cavalieri principle. At low magnification (2.5×), a systematic point grid was superimposed onto each Nissl-stained section using stereological software (Carl Zeiss, Oberkochen, Germany). Grid points falling within the contours of the entire amygdala and its subregions (BLA and CeA) were counted.

The volume (V) was calculated using the formula:

$$V = t \times a(p) \times \Sigma P$$

where t represents the distance between sampled sections (0.6 mm), $a(p)$ is the area associated with each grid point (0.02 mm^2), and ΣP is the total number of grid points counted for each structure per animal.

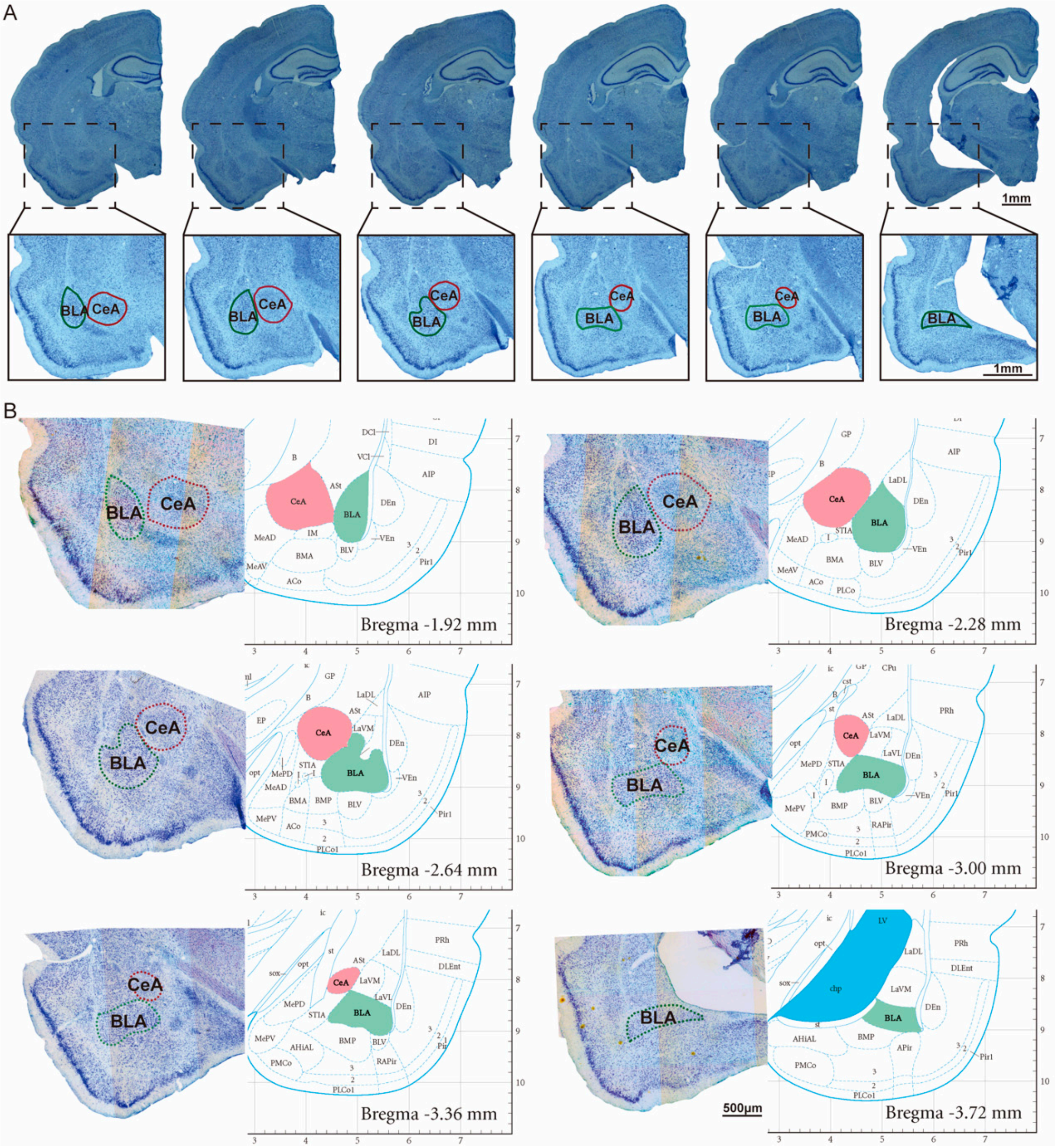

Figure 2. Cytoarchitectonic delineation and stereological volume estimation of the amygdala using Nissl staining. (A) Representative serial coronal sections illustrating the anterior–posterior extent of the amygdala. Nissl-stained images are shown at low (1.25×) and higher (2.5×) magnification, with BLA (green) and CeA (red) outlined. Scale bar: 1 mm. (B) Cytoarchitectonic criteria used for the delineation of amygdala subregions. The six panels correspond one-to-one to the six sections shown in (A). For each level (Bregma −1.92 mm to −3.72 mm), a representative higher-magnification Nissl-stained image (10×; left) is shown to visualize neuronal cell bands and regional differences in neuronal density. These cytoarchitectonic features were compared with the corresponding coronal levels in a standard rat brain atlas (Paxinos and Watson, *The Rat Brain in Stereotaxic Coordinates*; atlas interval 0.36 mm; right) to accurately define the boundaries between the BLA (green) and CeA (red). Scale bar: 500 µm. Solid and dashed lines are used only for visual guidance and do not indicate different experimental conditions.

Immunohistochemistry

Prior to immunostaining, free-floating sections were washed three times (10 min each) in 0.01 M phosphate-buffered saline (PBS, pH 7.4) to remove residual cryoprotectant. Sections were then incubated in PBS containing 0.3% Triton X-100 and 0.1% Tween-20 (PBS+T) for permeabilization. Unless otherwise noted, all washing and incubation steps were perfomed in 6-well Netwell plates at room temperature (RT, 21–23 °C) under gentle

agitation. Sections were blocked in PBS+T supplemented with 1% fetal bovine serum (FBS) and 5% normal goat serum (SP 9001-A) at 37 °C for 2 h to reduce nonspecific binding. Subsequently, sections were incubated with rabbit anti-glial fibrillary acidic protein (GFAP) primary antibody (ab7260, Abcam; 1:1000) at 4 °C for 72 h. After extensive rinsing in PBS+T, sections were incubated with biotinylated goat anti-rabbit IgG secondary antibody (SP 9001-B; 1:20) at 4 °C for 24 h, followed by incubation with biotin–HRP–streptavidin complex (SP 9001-C) at 37 °C for 4 h. Immunoreactivity was visualized using 3,3′-diaminobenzidine (DAB; ZLI-9018) as the chromogen, with color development monitored under a microscope (approximately 10 min). After termination of the reaction, sections were mounted onto gelatin-coated slides, dehydrated through a graded ethanol series, cleared in xylene, and coverslipped. Sections were examined throughout their thickness to confirm complete antibody penetration.

Stereological Analysis

The numbers of GFAP-positive astrocytes in the basolateral amygdala (BLA) and central amygdala (CeA) were estimated using the optical fractionator method. Amygdala subregions were delineated at low magnification (2.5×) based on Nissl-defined cytoarchitectonic criteria using a ZEISS stereology system (Carl Zeiss, Oberkochen, Germany). Sampling was performed in a systematic random manner to ensure that each counting site had an equal probability of being selected. Stereological counting was conducted using optical disector frames that were randomly and systematically distributed across each region of interest with an area sampling fraction (asf) of 15%. Immunohistochemically stained sections were used for analysis. A guard zone of 3 μm was applied at the upper surface of each section to avoid lost caps, and GFAP-positive astrocyte nuclei were counted within the subsequent 15-μm disector height. Only cells coming into focus within the disector height and meeting unbiased counting frame criteria were included, i.e., cells located entirely within the counting frame or intersecting only the inclusion line. The total number of astrocytes (N) was estimated according to the following formula:

$$N = \Sigma Q^{-} \times (1/ssf) \times (1/asf) \times (1/hsf)$$

where ΣQ^{-} represents the total number of GFAP-positive cells counted, ssf is the section sampling fraction, asf is the area sampling fraction, and hsf is the height sampling fraction. All delineation and counting procedures were performed under blinded conditions with respect to experimental group.

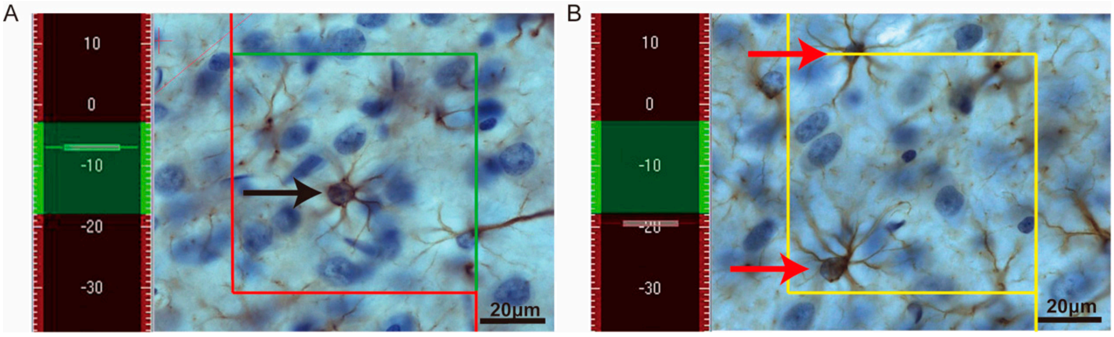

Figure 3. Unbiased counting rules for stereological quantification of GFAP-positive astrocytes. (A) Representative high-magnification image illustrating the application of an unbiased counting frame for stereological analysis of GFAP-positive astrocytes. The green line denotes the inclusion boundary, whereas the red lines and their extensions indicate the exclusion boundaries. A GFAP-positive astrocyte nucleus indicated by the black arrow was included in the count, as it was located entirely within the counting frame or intersected only the inclusion line. (B) Representative image showing GFAP-positive astrocytes excluded from stereological counting. Astrocyte nuclei indicated by red arrows were not counted because they intersected the exclusion lines. Scale bar = 20 μm.

Immunofluorescence

Following standard rinses in 0.01 M PBS, free-floating sections were processed for immunofluorescence staining using two distinct protocols. Unless otherwise stated, all washing and incubation steps were performed with gentle agitation. For GFAP/BrdU double immunofluorescence, sections were blocked with 5% normal goat serum diluted in PBS containing 0.3% Triton X-100. Sections were first incubated with anti-GFAP primary antibody at 4 °C for 48 h. To expose BrdU epitopes, sections were subsequently pretreated with HCl, neutralized with borate buffer, and then incubated with anti-BrdU antibody (ab6326, Abcam; 1:500) at 4 °C for 24 h. For GFAP/PSD95 double immunofluorescence, sections were blocked with 10% normal goat serum and simultaneously incubated with anti-GFAP (sc-33673, 1:1000) and anti-PSD95 (Cell Signaling Technology, #3450, 1:500) primary antibodies at 4 °C for 24 h. After primary antibody incubation, sections were thoroughly rinsed in PBS and incubated with the appropriate DyLight-conjugated secondary antibodies (DyLight 488 or 549; Abbkine; 1:200) at 37°C for 2 hours in the dark. Following final washes, sections were mounted with antifade mounting medium and imaged using a laser scanning confocal microscope (Andor, UK).

Astrocyte Morphological Complexity Analysis

Astrocyte morphological complexity was quantified using Fiji-ImageJ (NIH) following established skeleton- and Sholl-based analysis pipelines. After GFAP immunofluorescence imaging, Z-stack images were imported into Fiji-ImageJ, and the GFAP single-channel signal was selected for analysis. Z-stacks were converted into two-dimensional images using maximum intensity projection (Stacks → Z Project → Max Intensity). Image brightness and contrast were adjusted uniformly across all images to enhance signal-to-noise ratio while preserving structural integrity. The projected images were then binarized (Process → Binary) to extract astrocytic processes, followed by skeletonization (Process → Skeletonize) to generate single-pixel–wide representations of astrocyte processes.

To ensure representative sampling while avoiding pseudoreplication, astrocytes were selected using a systematic random sampling strategy. For each animal, three serial coronal sections containing the basolateral amygdala (BLA) and central amygdala (CeA) were analyzed. Within each section, three non-overlapping fields of view were randomly selected per subregion, and one GFAP-positive astrocyte with a clearly identifiable soma and intact processes was randomly chosen from each field for analysis. Thus, a total of 18 astrocytes were analyzed per animal, and measurements were first averaged within each animal. The animal was treated as the statistical unit for all subsequent analyses.

Skeletonized images were analyzed using the Analyze Skeleton plugin to extract quantitative parameters of astrocytic branching architecture, including maximum branch length and number of endpoints. Maximum branch length was defined as the longest individual process within a single astrocyte, and endpoints were defined as terminal points of the skeletonized processes. To further assess astrocytic process complexity and spatial organization, Sholl analysis was performed using the Sholl Analysis plugin, with concentric circles generated outward from the center of the astrocyte soma at 3 μm intervals. The total number of intersections across all radii was calculated for each astrocyte and used as an index of morphological complexity. The overall workflow of astrocyte morphological analysis is illustrated schematically in Figure 7A.

Astrocyte–PSD95 Colocalization Analysis

Astrocyte–synapse colocalization was quantified using Imaris software (Bitplane, Oxford Instruments) based on confocal Z-stack images acquired under identical imaging settings across all experimental groups. Raw Z-stack images were imported into Imaris, and brightness and contrast were adjusted uniformly for visualization purposes only.

Three-dimensional reconstructions of individual astrocytes were generated from the GFAP signal using the Surface module, ensuring complete coverage of astrocytic

processes and preservation of their native spatial architecture. $PSD95^+$ puncta were identified using the Spots module based on fluorescence intensity and spatial distribution. To minimize background noise, only puncta with an estimated diameter ≥ 0.6 μm were included in the analysis.

Astrocytic surfaces and $PSD95^+$ puncta were spatially overlaid in three dimensions. A colocalization event was defined as direct spatial contact between the astrocytic surface and a $PSD95^+$ punctum. The number of astrocyte-associated $PSD95^+$ puncta was quantified on a per-cell basis.

For visualization, three-dimensional reconstructions of $PSD95^+$ puncta were generated while preserving the original spatial dimensions of astrocytic processes and synaptic structures. Quantitative analyses were performed exclusively on raw image data using identical acquisition and thresholding parameters across all experimental groups.

Single-cell measurements were averaged within each animal, and statistical analyses were conducted using the animal as the unit of analysis. All analyses were performed under blinded conditions.

Normalization analysis of astrocyte-associated PSD95+ puncta

Because changes in astrocyte volume may influence the absolute number of PSD95+ puncta associated with each astrocyte, a normalization analysis was performed. For each reconstructed astrocyte, the number of astrocyte-associated PSD95+ puncta was divided by astrocyte volume to obtain normalized PSD95+ puncta density (puncta/μm^3). These normalized values were then used for group comparisons and statistical analyses.

References

1. Luo, Y.; Xiao, Q.; Wang, J.; Jiang, L.; Hu, M.; Jiang, Y.; Tang, J.; Liang, X.; Qi, Y.; Dou, X.; et al. Running Exercise Protects Oligodendrocytes in the Medial Prefrontal Cortex in Chronic Unpredictable Stress Rat Model. Translational Psychiatry 2019 9:1 2019, 9, 322-, doi:10.1038/s41398-019-0662-8.
2. Willner, P. The Chronic Mild Stress (CMS) Model of Depression: History, Evaluation and Usage. Neurobiol. Stress 2017, 6, 78–93, doi:10.1016/j.ynstr.2016.08.002.
3. Fu, Q.; Qiu, R.; Chen, L.; Chen, Y.; Qi, W.; Cheng, Y. Music Prevents Stress-Induced Depression and Anxiety-like Behavior in Mice. Translational Psychiatry 2023 13:1 2023, 13, 317-, doi:10.1038/s41398-023-02606-z.
4. Gundersen, H.J.G.; Jensen, E.B. The Efficiency of Systematic Sampling in Stereology and Its Prediction*. J. Microsc. 1987, 147, 229–263, doi:10.1111/J.1365-2818.1987.TB02837.X.
5. Tantiwisawaruji, S.; Rocha, M.J.; Silva, A.; Pardal, M.A.; Kovitvadhi, U.; Rocha, E. A Stereological Study of the Three Types of Ganglia of Male, Female, and Undifferentiated Scrobicularia Plana (Bivalvia). Animals 2022, Vol. 12, 2022, 12, doi:10.3390/ANI12172248.
