# Supplementary material for: Running Exercise Promotes Astrocyte-Mediated Structural Plasticity in the Amygdalar BLA and CeA to Alleviate Anhedonia-like Behavior Alterations
Source: Cells. 2026 Apr 14;15(8):693. doi: 10.3390/cells15080693 (PMC13114546; doi:10.3390/cells15080693)
Supplement: Supplementary file 1 [file cells-15-00693-s001.zip › Supplementary Table S4.pdf]

Supplementary Table S4. Results of the open field test.

	Control group (n=15)	CUS group (n=15)	CUS+running group (n=15)
Total distance (×10^3 cm)	3.25±1.98	3.27±1.74	3.20±7.44
Time spent in center (s)	4.38±9.68	8.13±11.25	18.22±19.79
Percentage of time spent in center (%)	0.007±0.016	0.014±0.019	0.030±0.033
Distance traveled in center（cm）	34.26±54.94	59.67±67.77	138.31±178.67
Percentage of distance traveled in the center (%)	0.99±1.46	1.87±1.96	3.82±4.55
Average speed (cm/s)	5.41±3.30	5.46±2.91	5.34±1.24

Table note: Data are presented as mean ± SD (n = 15 per group). Statistical comparisons were performed using one-way ANOVA.
