# Supplementary material for: Running Exercise Promotes Astrocyte-Mediated Structural Plasticity in the Amygdalar BLA and CeA to Alleviate Anhedonia-like Behavior Alterations
Source: Cells. 2026 Apr 14;15(8):693. doi: 10.3390/cells15080693 (PMC13114546; doi:10.3390/cells15080693)
Supplement: Supplementary file 1 [file cells-15-00693-s001.zip › Supplementary Table S5.pdf]

Supplementary Table S5. Stereological estimates of BLA and CeA volumes and measures of sampling variability.

	Control	CUS	CUS+running
**Number of sections sampled**			
	7-9	8-9	7-9
**Volume (mm^3)**			
BLA	1.03±0.06	1.05±0.11	1.09±0.14
CeA	0.86±0.03	0.83±0.10	1.04±0.06
**Observed coefficient of variation (OCV)**			
BLA	0.061	0.102	0.129
CeA	0.038	0.24	0.059
**Observed coefficient of error (OCE)**			
BLA	0.012	0.011	0.011
CeA	0.012	0.011	0.011
**OCE^2/OCV^2**			
BLA	0.039	0.011	0.007
CeA	0.099	0.002	0.033

Table note: Volumes are presented as mean ± SD. Abbreviations: BLA, basolateral amygdala; CeA, central amygdala; OCE, observed coefficient of error; OCV, observed coefficient of variation.
