# Supplementary material for: Running Exercise Promotes Astrocyte-Mediated Structural Plasticity in the Amygdalar BLA and CeA to Alleviate Anhedonia-like Behavior Alterations
Source: Cells. 2026 Apr 14;15(8):693. doi: 10.3390/cells15080693 (PMC13114546; doi:10.3390/cells15080693)
Supplement: Supplementary file 1 [file cells-15-00693-s001.zip › Supplementary Table S1.pdf]

Supplementary Table S1: Schedule of the CUS paradigm.

Time	Monday	Tuesday	Wednesday	Thursday	Friday	Saturday	Sunday
Week 1	Strobe light	Cage tilting	Water deprivation	Noise	Continuous lighting	Food deprivation	BWT
	Electric foot shock	Damp bedding	Continuous darkness during daytime	Tail clamping	Restraint	Intermittent illumination	
Week 2	Water deprivation	Cage tilting	Continuous lighting	Cold stress	Empty bottle exposure	Intermittent illumination	BWT
	Electric foot shock	Damp bedding	Cage shaking	Strobe light	Continuous darkness during daytime	Restraint	
Week 3	Strobe light	Food deprivation	Water deprivation	Intermittent illumination	Cage tilting	Continuous darkness during daytime	BWT
	Tail clamping	Continuous darkness during daytime	Hot stress	Noise	Bedding removed	Restraint	
Week 4	Cage tilting	Water deprivation	Empty bottle exposure	Food deprivation	Continuous lighting	Intermittent illumination	BWT
	Bedding removed	Restraint	Electric foot shock	Strobe light	Cage shaking	Cold stress	
Week 5	Food deprivation	Continuous lighting	Water deprivation	Cage tilting	Continuous darkness during daytime	Intermittent illumination	SPT
	Tail clamping	Noise	Hot stress	Damp bedding	Restraint	Cage shaking	BWT
